# Silencing the *cyp314a1* and *cyp315a1* Genes in the *Aedes albopictus* 20E Synthetic Pathway for Mosquito Control and Assessing Algal Blooms Induced by Recombinant RNAi *Microalgae*

**DOI:** 10.3390/insects16101033

**Published:** 2025-10-07

**Authors:** Xiaodong Deng, Changhao He, Chunmei Xue, Dianlong Xu, Juncai Li, Xiaowen Fei

**Affiliations:** 1 Department of Biochemistry and Molecular Biology & Key Laboratory of Tropical Translational Medicine of Ministry of Education, College of Basic Medical Sciences, Hainan Medical University, Haikou 571199, China; 2 Institute of Tropical Bioscience and Biotechnology, Chinese Academy of Tropical Agricultural Science & Key Laboratory of Biology and Genetic Resources of Tropical Crops of Hainan Province, Hainan Institute for Tropical Agricultural Resources, Haikou 571101, China; 3 College of Life Sciences and Agriculture, Jiamusi University, Jiamusi 154007, China; 4 Hainan Provincial Key Laboratory for Functional Components Research and Utilization of Marine Bio-Resources, Haikou 571101, China; 5 Zhanjiang Experimental Station, Chinese Academy of Tropical Agricultural Science, Zhanjiang 524013, China

**Keywords:** CYP314A1 and CYP315A1, *Chlamydomonas reininatus*, *Chlorella*, RNAi, *Aedes albopictus*, 18S rDNA and 16S rDNA high-throughput sequencing

## Abstract

As one of the key vectors for the transmission of Dengue fever, *Aedes albopictus* is highly ecologically adaptable. The development of environmentally compatible biological defence and control technologies has therefore become an urgent need for vector biological control worldwide. This study constructed and used double-stranded RNA (dsRNA) expression vectors targeting the *cyp314a1* and *cyp315a1* genes of *Ae. albopictus* to transform *Chlamydomonas reinhardtii* and *Chlorella vulgaris*, achieving RNA interference (RNAi)-mediated gene silencing. The efficacy of the RNAi recombinant algal strain biocide against *Ae. albopictus* was evaluated by administering it to *Ae. albopictus* larvae. The results showed that the oral administration of the *cyp314a1* and *cyp315a1* RNAi recombinant *C. reinhardtii*/*C. vulgaris* strains was lethal to *Ae. albopictus* larvae and severely affected their pupation and emergence. The recombinant algal strains triggered a burst of ROS (Reactive Oxygen Species) in the mosquitoes’ bodies, resulting in significant increases in the activities of the superoxide dismutase (SOD), peroxiredoxin (POD) and catalase (CAT), as well as significant upregulation of the mRNA levels of the CME pathway genes in larvae. In the simulated field experiment, the number of *Ae. albopictus* was reduced from 1000 to 0 in 16 weeks by the RNAi recombinant *Chlorella*, which effectively controlled the population of mosquitoes. Meanwhile, the levels of nitrogen (N), phosphorus (P), nitrate, nitrite, ammonia and COD (Chemical Oxygen Demand) in the test water decreased significantly. High-throughput sequencing analyses of 18S rDNA and 16S rDNA showed that, with the release of RNAi recombinant *Chlorella* into the test water, the biotic community restructuring dominated by resource competition caused by algal bloom, as well as the proliferation of anaerobic bacteria and the decline of aerobic bacteria triggered by anaerobic conditions, are the main trends in the changes in the test water. This study is an important addition to the use of RNAi recombinant microalgae as a biocide.

## Simple Summary

This study explores a novel approach to controlling *Aedes albopictus*, a key vector for dengue fever, by using RNA interference (RNAi) mediated by genetically modified microalgae. We constructed double-stranded RNA (dsRNA) expression vectors that target the *cyp314a1* and *cyp315a1* genes of *Ae. albopictus*, and we transformed two species of algae, *Chlamydomonas reinhardtii* and *Chlorella vulgaris*, to express these dsRNAs. The recombinant RNAi algal strains were then fed to *Ae. albopictus* larvae, resulting in high mortality rates, disrupted pupation and reduced emergence. The algae triggered an increase in reactive oxygen species (ROS) and enzyme activity in the larvae, indicating redox stress. In simulated field experiments, RNAi recombinant *Chlorella* significantly reduced mosquito populations, and improved water quality by reducing nitrogen, phosphorus and others. Furthermore, with the release of RNAi recombinant *Chlorella* into the test water, the biotic community restructuring dominated by resource competition caused by algal bloom, as well as the proliferation of anaerobic bacteria and the decline of aerobic bacteria triggered by anaerobic conditions, are the main trends in the changes in the test water. This study is an important addition to the use of RNAi recombinant microalgae as a biocide.

## 1. Introduction

Dengue fever is an acute arboviral disease primarily transmitted through *Aedes* mosquito bites [1]. According to World Health Organization (WHO) surveillance data (2023 update), reveal a dramatic tenfold increase in reported cases, from 500,000 in 2000 to 5.2 million in 2023. This increase has been accompanied by over 5000 dengue-related fatalities, representing the highest mortality figures documented since systematic monitoring began [2,3,4].

The primary vectors of Dengue fever are *Aedes* mosquitoes, notably *Ae. aegypti* and *Ae. albopictus*. These species alsotransmit Zika virus disease, Yellow fever, Rift Valley fever, and Chikungunya fever [5,6,7]. *Ae. albopictus*, in particular, demonstrates high ecological plasticity and has become the main dengue vector in China. It is distributed across a wide range of climates, from temperate regions (up to 45° north latitude) to tropical ecosystems.

Using biotechnological interventions to suppress vector populations represents an inevitable paradigm shift in the control of mosquito-borne diseases. Current advancements focus on two main strategies: Wolbachia-based population replacement and transgenic mosquito approaches that induce male sterility through genetic modification [8,9,10,11,12]. However, scalability remains challenging due to the substantial costs associated with the mass rearing of mosquitoes and the sex sorting protocols required for field implementation [13,14]. Microalgae-mediated biocontrol is a promising alternative that leverages natural trophic relationships in mosquito larval habitats. Microalgae such as *Chlorella* spp., *Scenedesmus* spp., *Spirulina* spp. and *Chlamydomonas* spp. are essential dietary components for developing *Aedes* larvae, and can be cultivated on a large scale at low cost. These microalgae offer distinct operational advantages. Compared to Wolbachia-infected or transgenic male-sterile *Ae. aegypti* strains, algal-based interventions are more economically feasible and easier to control environmentally. Strategic deployment in contained aquatic ecosystems enables rapid algal dominance, thus gradually reducing the number of Aedes mosquitoes in the area. This novel approach to vector suppression is particularly promising in interrupting the transmission networks of arboviruses such as dengue, Zika and yellow fever.

We have used microalgae expressing RNA interference fragments of the Aedes *HR3* (*Hormone receptor 3*), *3HKT* (*3-Hydroxykynurenine transaminase*) and *CHSA* (*Chitin synthase A*) genes to effectively control *Aedes* populations [15,16,17]. However, it has been shown that prolonged selection pressure for silencing against specific gene targets leads to tolerance (drug resistance) in mosquitoes to the targeted RNAi-interfering algal strains [18,19,20,21]. Cytochrome P450 (CYP450) is an ancient and widely functional superfamily of monooxygenases [22]. The genes encoding these enzymes are among the largest gene superfamilies in plants, animals and fungi and are essential for development, growth, and reproduction [23,24]. CYP450 are present in almost all organisms, including some viruses, with a few exceptions, such as *Escherichia coli*. Insects possess only four CYP450 clades, which is significantly fewer than the ten found in humans [25,26]. The functions of CYP450 in insects are diverse and include metabolic detoxification, growth and development regulation, and environment adaptation. In metabolic detoxification, insects adapt to the chemical defences of host plants by catalysing the hydroxylation or oxidation of toxic plant xenobiotics to produce less toxic or non-toxic compounds via CYP450 [27,28,29,30,31,32,33,34,35]. CYP450 can also degrade insecticides [36,37]. For instance, RNAi silencing of the MusiDN2722 CYP450 gene in *Megalurothrips usitatus* significantly increased susceptibility to acetamiprid, confirming the direct role of CYP450 in insecticide detoxification [38]. Additionally, insect CYP450s participate in the biosynthesis of ecdysone to regulate larval moulting, metamorphosis, and embryonic development [39,40,41,42]. These CYP450s include neverland (CYP307A1), spookier (CYP307A2), phantom (CYP306A1), disembodied (CYP302A1), shadow (CYP315A1), and shade (CYP314A1). CYP315A1 and CYP314A1 jointly catalyse the final conversion of cholesterol into the physiologically active 20-hydroxyecdysterone (20E) [43,44]. Given the crucial role of ecdysone in inset development and metamorphosis, this study selected the key enzymes CYP314A1 and CYP315A1 in the 20E anabolic pathway of *Aedes* mosquitoes as RNAi targets. The dsRNAs of these enzymes were then recombined into the genomes of *Chlorella vulgaris* and *Chlamydomonas reinhardtii*, and the microalgae were fed to *Aedes* mosquito larvae in order to inhibit the growth of *Aedes* mosquito populations.

This study aims to utilise low-cost microalgae by releasing recombinant strains into waters adjacent to mosquito breeding sites to control local mosquito populations, while concurrently assessing the environmental impact of algal blooms induced by these engineered microalgae. However, due to governmental regulations on genetically modified organisms (GMOs), the direct release of recombinant microalgae into natural water bodies (e.g., ponds and lakes) was prohibited. Consequently, we established a contained experimental area that simulated wild-type aquatic conditions in order to replicate microalgal growth and reproduction dynamics, while also monitoring of mosquito population numbers. From September 2022 to June 2023, we systematically monitored phytoplankton, zooplankton, and prokaryotic communities in the simulated aquatic environment using water quality assays and 18S and 16S rDNA high-throughput sequencing. The population densities and structural profiles of aquatic organisms were analysed under *Chlorella* bloom conditions. These findings provide foundational data for the future practical application of recombinant microalgae in the control of mosquito populations and the prevention of mosquito-borne diseases such as dengue fever.

## 2. Methods

### 2.1. Bioinformatic Analysis

The CYP314A1 and CYP315A1 proteins in *Ae. albopictus* and other organisms were identified using the *Ae. albopictus* CYP314A1 and CYP315A1 protein sequence as seed sequences in BLASTP (Version 2.13.0, Bethesda, MA, USA) searches of the Vectorbase (https://vectorbase.org/vectorbase/app, accessed on 7 May 2024) and NCBI (National Center for Biotechnology Information) (https://www.ncbi.nlm.nih.gov/, accessed on 7 May 2024) databases. An online InterProScan analysis (https://www.ebi.ac.uk/interpro/search/sequence/, accessed on 16 April 2025) was carried out to identify the domains of the CYP314A1 and CYP315A1 proteins. A phylogenetic tree was then constructed using MEGA11 software (Version 11, Temple University, Philadelphia, PA, USA) based on the alignment of the CYP314A1 and CYP315A1 orthologue in *Ae. albopictus* and other organisms. The tree was generated using the neighbour-joining (NJ) method with Poisson correction and pairwise deletion parameters. Based on sequence information obtained from online databases, a schematic representation of CYP314A1 and CYP315A1 homologous protein structures was created. To identify CYP450 homologous proteins, conserved domain sequences of *Ae. albopictus* CYP450, including WxxxR (Helix-C), GxE/DTT/S (Helix-I), ExxR (Helix-K), PxxFxPE/DRF (PERF), and PFxxGxRxCxG/A (heme-binding domain), were queried in the PFAM (http://pfam.xfam.org/, accessed on 16 April 2025), NCBI and VactorBase (https://vectorbase.org/vectorbase/app, accessed on 15 April 2025) databases After filtering out redundant, 100% identical transcripts from the protein-coding sequence, those encoding at least 300 amino acids were retained. All CYP450 amino acid sequences were collected and annotated using InterPro, and multiple sequences were aligned using ClustalW (Version 11, Dublin, Ireland) and MEGA11. The JTT model was selected for the above processed sequences using maximum likelihood tree. After constructing the tree, iTOL was used to classify it by clan.

### 2.2. Mosquito Maintenance

Mosquitoes rearing was performed as previously reported [15,16,17]. Local wild type *Ae. albopictus* were captured in Haikou, China, and propagated for five generations in the laboratory, which were kept in an insectary at the Chinese Academy of Tropical Agricultural Science. The mosquitoes were kept at a temperature of 25 °C and a relative humidity of 80%, with a 12 h cycle of darkness and light. The mosquito cages were also equipped with a sponge saturated with sugar water to provide nourishment. Chicken blood was used to feed the female mosquitoes. Following oviposition on moist filter paper, the eggs were harvested and kept in a dry state. The eggs were then transferred to incubation water containing rat food to facilitate hatching.

### 2.3. Microalgae Strains and Plasmids

The *Chlorella vulgaris* HOC5 strain was isolated from local waters in Hainan, China, and cultured in TAP medium. *Chlamydomonas reinhardtii* CC425 was purchased from the Freshwater Algae Culture Collection at the Institute of Hydrobiology (FACHB), Chinese Academy of Sciences. Liquid cultures were maintained at 25 °C in a shaker at 180 rpm under continuous light (150 µmol/(m^2^·s)) [16]. Plasmid pMD18-T was constructed locally by our research group and stored in our laboratory. The pMaa7 IR/XIR RNAi expression vector was purchased from the *Chlamydomonas* centre at Duke University in Durham, NC, USA.

### 2.4. Construction of RNAi Vectors

Total RNA was extracted from *Ae. albopictus* larvae using TRIzol reagent (Takara, Shiga, Japan) according to the manufacturer’s protocol [15]. Complementary DNA (cDNA) was then synthesised from the total RNA using oligo-dT primers (Shenggong, Shanghai, China) and served as the template for PCR amplification. The *cyp314a1* (AALFPA_063667) and *cyp315a1* (AALFPA_079547) DNA fragment were PCR-amplified using the primers listed in Supplementary Table S1, respectively. These primers target the corresponding *cyp314a1* and *cyp315a1* CDS regions from 390 to 805 and 5 to 324, respectively. The amplified fragments were then inserted into the pMD18-T plasmid backbone (pMD-cyp314a1/cyp315a1). These fragments were then digested with HindIII/BamHI and XbaI/SalI, before being inserted into the corresponding cloning sites of the pT282 plasmid, which contained the inverted repeat sequence of the respective genes. The RNAi (pMaa7 IR/XIR) and intermediate (pT282-cyp314a1/cyp315a1) vectors were both digested using EcoRI and then ligated to produce the RNAi recombinant vectors, pMaa7 IR/cyp314a1IR and pMaa7 IR/cyp315a1IR [45].

### 2.5. Microalgae Transfection

The *Chlamydomonas*/*Chlorella* was grown, collected by centrifugation, and resuspended in electroporation buffer. For transfection, 2 µg of either pMaa7 IR/cyp314a1IR or pMaa7 IR/cyp315a1IR was mixed with the 100 µL recipient *Chlamydomonas*/*Chlorella* cells (2 × 10^3^ cells µL^−1^) and the mixture was then incubated on ice for 15 min. The mixture was then transferred into an electroporation cuvette and subjected to an electric pulse in an electrometer (BTX, ECM 630, Shelton, WA, USA) with a 1600 V/cm voltage and a pulse time of 1 ms. The suspension was then transferred to TAP medium containing 60 mmol/L sorbitol and allowed to recover overnight. Finally, the cells were harvested by centrifugation at 10,000× *g* for 3 min and plated on TAP agar plates containing 10 µg/mL paromomycin. The plates were incubated at 23 °C under continuous illumination of 100 µmol m^−2^ s^−1^. Algal colonies appeared after 5–10 days [46,47].

### 2.6. Mosquito Feeding Experiments

After the RNAi vector, pMaa7IR/cyp314a1IR and pMaa7IR/cyp315a1IR had been transferred into *Chlamydomonas*/*Chlorella*. The transgenic microalgae were characterised using PCR to determine if the *cyp314a1* or *cyp315a1* inverted repeat expression cassette had been integrated into the *Chlamydomonas*/*Chlorella* genome. The positive clones were used to feed the mosquitoes in the laboratory. As previously described, ten and 300 mosquito feeding experiments were performed in the insectary [15,16]. In the 10-mosquito feeding experiment, the mosquitoes were divided into experimental and control groups. Each group contained 10 L1 larvae in 5 mL water supplemented with 75 mg fresh microalgae. The larvae in the experimental groups were fed the *cyp314a1* and *cyp315a1* RNAi transgenic *Chlamydomonas*/*Chlorella* strains, respectively. In contrast, the larvae in the control groups were fed *C. reinhardtii* CC425 or *C. vulgaris* HOC5, water, feed, and the empty plasmid pMaa7IR/XIR transgenic strain. The experiments were performed in triplicate, recording the mortality, pupation, and adult emergence rates.

In the 300-mosquito feeding experiment, 300 L1 larvae were placed into 50 mL of water supplemented with 750 mg of fresh microalgae. The larvae feeding the recombinant *Chlamydomonas*/*Chlorella* were set as the test treatments, whereas the larvae fed with *C. reinhardtii* CC425 or *C. vulgaris* HOC5, water, and feed were used as controls. The triplicate experiments included records of mortality, pupation, and adult emergence rates.

To measure body size, five third-instar mosquito larvae were randomly selected from each treatment and control group. Larva was placed a stereomicroscope (Keyence VHX-1000 Super Depth 3D Stereomicroscope, Osaka, Japan), and body length and width were measured using the built-in scale. Body width was determined at the widest segment, and body length was measured from the anterior margin of the head to the tip of the abdomen. The values represented are the mean measurements from the five larvae.

### 2.7. Measurement of Larval Superoxide Dismutase (SOD), Peoxidase (POD) and Catalase (CAT) Activities

The larvae were fed either *C. reinhardtii* CC425 or *C. vulgaris* HOC5 (used as controls). Some larvae were also fed recombinant *Chlamydomonas* CC425-CYP314A1 and CC425-CYP315A1, while others were fed recombinant *Chlorella* HOC5-CYP314A1 and HOC5-CYP315A1. Ten to fifteen L4 larvae were sampled from each group, mixed with 5 mmol/L phosphate-buffer solution (pH 7.8, Shenggong, Shanghai, China), ground and homogenised on ice, then centrifuged at 10,000× *g* for 20 min at 4 °C. The soluble protein content was determined using the G-250 method (Bradford Protein Concentration Assay Kit, Beyotime Biotechnology, Shanghai, China). SOD activity was measured using an SOD activity assay kit (Beyotime Biotechnology, Shanghai, China), POD activity was measured using a peroxidase (POD) activity assay kit (Beijing Solarbio Technology Co., Ltd., Beijing, China), and CAT activity using a catalase assay kit (Beyotime Biotechnology, Shanghai, China) [48,49,50,51].

### 2.8. The 20-Hydroxyecdysone(20E) Compensation Test

A total of six groups were tested. In each group, 30 *Ae. albopictus* larvae were fed a 15 mg/mL algal solution. 300 µL of 20E (1 mg/mL) was added to the relevant groups, and the mosquitoes were fed for 15 days [52,53]. Mosquitoes fed *C. reinhardtii* CC425 and *C. reinhardtii* CC425+20E were used as control groups. The treatment groups comprised mosquitoes fed *cyp314a1* and *cyp315a1* RNAi transgenic *Chlamydomonas* lines and mosquitoes fed *cyp314a1* and *cyp315a1* RNAi transgenic *Chlamydomonas* lines with the addition of 20E. The mortality rate, pupation rate and eclosion rate were recorded for each group. Total RNA was extracted from L4 larvae, and the expression levels of the relevant target genes (*cyp314a1* and *cyp315a1*) were detected in the larvae by RT-qPCR. The experiment was repeated three times.

### 2.9. RT-qPCR

For the reverse transcription quantitative polymerase chain reaction (PCR), 20–30 L3 larvae were collected and pooled from each treatment. Total RNA was isolated from the larvae using TRIzol reagent (Takara, Shiga, Japan), following the manufacturer’s instructions [54]. Single-stranded cDNA was synthesised from the total RNA using oligo-dT primers (Shenggong, Shanghai, China). Real-time PCR was performed using a BioRad iCycler iQ Real-Time PCR Detection System (Bio-Rad, Hercules, CA, USA) and SYBR Green as a fluorescent dye. Primers targeting the *Aedes* RPS17 gene were used as internal controls [55]. The primers used to amplify the *cyp314a1* and *cyp315a1* target genes, as well as the primers used to amplify the genes involved in the clathrin-mediated endocytosis (CME) pathway, are listed in the Supplementary Table S1. The amplification rate (Ct) of each transcript was calculated using the PCR baseline subtraction method and iCycler software (version 2.3, Bio-Rad, Hercules, CA, USA) (constant fluorescence level). The cycle threshold (Ct) was determined in triplicate and the relative fold differences were calculated using the relative quantification method (2^−ΔΔCT^) [56]. Gene expression was determined relative to the endogenous control.

### 2.10. Simulated Field Trials

Simulated field trials were performed as described in the previous study [16]. An unused factory building on the outskirts of Haikou City was chosen for the experiment. The mosquitoes were reared in polyester mesh cages with a mesh size of 0.4 mm and a capacity of 1.5 m^3^ [57]. Four 1000 L buckets for microalgae production were placed in the cages, which were fitted with LED lights. An aeration pump was used to ensure constant water circulation. The buckets were then filled with 10 L of cultivated microalgae. From 10 L to 200 L, sufficient light and aeration were maintained for the *Chlorella* culture. Jinniu Ling reservoir water was then added to bring the volume to 800 L, and the incubation process continued until the concentration of *Chlorella* cells reached 2 × 10^6^–2 × 10^7^ cells/mL. Around 1000 L1 larvae were placed in each bucket. A 10% sucrose solution was prepared for the male adults, while the egg-laying females were given chicken blood. The number of adult Aedes mosquitoes was counted weekly, in a similar manner to how the abundance of *Chlorella* in the water was determined. Blood and sugar water were discontinued one day prior to counting the mosquitoes to temporarily reduce their physical strength. Mosquito counting was conducted between 3:00 and 6:00 a.m. A dim LED flashlight was used to illuminate the cages during the procedure [16].

Water quality parameters were analysed in accordance with the Environmental Quality Standards for Surface Water (GB 3838-2002) [58]. Concentrations of ammonium nitrogen (NH_3_-N), nitrate (NO_3_^−^), nitrite (NO_2_^−^) and chemical oxygen demand (COD) were quantified using a Hach DR3900 spectrophotometer (Hach Company, Loveland, CO, USA). Nitrogen (N) levels were determined through alkaline persulfate digestion followed by UV spectrophotometric detection, while phosphorus (P) concentrations were measured using the ammonium molybdate spectrophotometric method [59,60].

### 2.11. Sample Preparation and DNA Extraction of the Test Water

An 18S and 16S rDNA high-throughput sequencing analysis was performed on the water from simulated-field trials to detect the effect of recombinant RNAi *Chlorella* on eukaryotic and prokaryotic organisms in the test water. After feeding *Ae. albopictus* for 30 days, a 1 L water sample was collected from the bucket containing the mosquitoes and filtered through a 0.22 μm polycarbonate membrane (Millipore, Burlington, MA, USA) under a 30 kPa vacuum to collect the plankton. The membranes were stored at −80 °C until analysis. Genomic DNA was extracted using a modified Winnepenninckx et al. method [61].

### 2.12. Amplification and Sequencing of the Hypervariable Region

The V4 hypervariable region of the 18S rDNA and the V3-V4 region of the 16S rDNA were selected as targets for high-throughput sequencing analysis. The target regions were amplified using the primers listed in Supplementary Table S1 [62,63]. For the 18S rDNA sequencing, sequence libraries were constructed using the NEBNext Ultra DNA Library Prep Kit for Illumina (San Diego, CA, USA) in accordance with the manufacturer’s protocol. The libraries were then sequenced on the Illumina HiSeq2500 platform. For 16S rDNA sequencing, the purified PCR products were sequenced on an Illumina MiSeq (Model MiSeq, Illumina, San Diego, CA, USA) platform. Data optimisation was conducted using the Chen and Jiang approach [64]. Low-quality sequences (i.e., those less than 200 base pairs in length) and sequencing primer adapters located at the 3′ ends of the primers were eliminated. After identifying chimeric sequences, high-quality sequences exhibiting 97% sequence identity were grouped into OTUs. The taxonomic categorisation of the OTUs was determined using USEARCH (version 5.2.236, Robert C. Edgar, San Francisco, CA, USA). Alpha diversity analysis was facilitated by calculating indices such as Shannon, Observed Species and Chao1 using QIIME software (version 1.9.1, University of Colorado, Boulder, CO, USA). Differences in community structure between samples or groups were analysed using principal coordinate analysis (PCoA) plots generated with R software (version 2.15.3, R Foundation for Statistical Computing, Vienna, Austria) [65,66].

### 2.13. Statistical Analyses

Data analysis was performed using the Statistical Package for the Social Sciences (SPSS, version 26.0, IBM, Chicago, IL, USA) and results are presented as the mean ± standard deviation (S.D.). Duncan’s multiple range test was performed to examine significant differences between means. Values with a *p*-value of less than 0.05 were considered statistically significant. Asterisks indicate statistical significance in all cases: * *p* < 0.05; ** *p* < 0.01. Error bars show the standard deviation [67]. The 95% confidence interval (CI) was calculated using IBM SPSS Statistics 26 (version 26.0, IBM, Chicago, IL, USA). For PERMANOVA F-test, differences in microbial community structure among treatments were assessed using a permutational multivariate analysis of variance based on Bray–Curtis dissimilarity matrices. Grouping assignment (treatments) was used as the explanatory factor. The F-statistic was derived by comparing between-group variance to within-group variance, and significance was determined through 999 permutations of sample labels [67].

## 3. Results

### 3.1. CYP314A1 and CYP315A1 Proteins

A BlastP search was performed using *Ae. albopictus* CYP314A1 and CYP315A1 as source sequences to retrieve a large number of amino acid sequences. Several representative CYP314A1 and CYP315A1 orthologues from different species were selected for further analysis (Supplementary Tables S2 and S3), including characterisation of the structural domains of the nucleic acids and proteins (Supplementary Figures S1 and S2) and phylogenetic analyses. Analysis of the CYP314A1/CYP315A1 protein sequences using the online tool Interproscan (https://www.ebi.ac.uk/interpro/search/sequence/, accessed on 16 April 2025) revealed that all p450 domains were present. Results from protein sequence alignment showed that all p450 domains of CYP314A1 and CYP315A1 contained the following functional regions: WxxxR (Helix-C), GxE/DTT/S (Helix-I), ExxR (Helix-K), PxxFxPE/DRF (PERF), and PFxxGxRxCxG/A (heme-binding domain) (Supplementary Figures S3 and S4). Analysis of the CYP450 gene family in *Ae. albopictus* revealed that the species contains 81 genes distributed among clan 2 (nine genes), clan 3 (52 genes), and clan 4 (27 genes), as well as the MITO family (seven genes). The genes involved in ecdysone synthesis belonged to clan 2 (e.g., *cyp306a1*, *cyp307a1*, and *cyp18a1*) and the MITO family (e.g., *cyp302a1*, *cyp315a1*, and *cyp314a1*) (Supplementary Figure S5 and Table S4).

### 3.2. Cyp314a1 and cyp315a1 RNAi Recombinant Chlamydomonas/Chlorella Are Lethal to Ae. albopictus

The target genes selected for RNAi were *cyp314a1* and *cyp315a1* (AALFPA_063667 and AALFPA_079547), which are key enzymes in the *Ae. albopictus* 20E synthesis pathway. The *cyp314a1* and *cyp315a1* RNAi interference fragments were amplified using *Ae. albopictus* cDNA as a template. The amplified fragments were approximately 416 bp and 319 bp long, respectively. These fragments were then cloned forward and reverse into the pMaa7IR/XIR vector to create the recombinant RNAi vectors pMaa7IR/cyp314a1IR and pMaa7IR/cyp315a1IR. These RNAi vectors were subsequently transformed into *C. reinhardtii* CC425 and *C. vulgaris* HOC5. Several positive recombinant algal strains were identified through the implementation of PCR, and these were then employed in subsequent experiments (Supplementary Figure S6).

**Figure 1:**
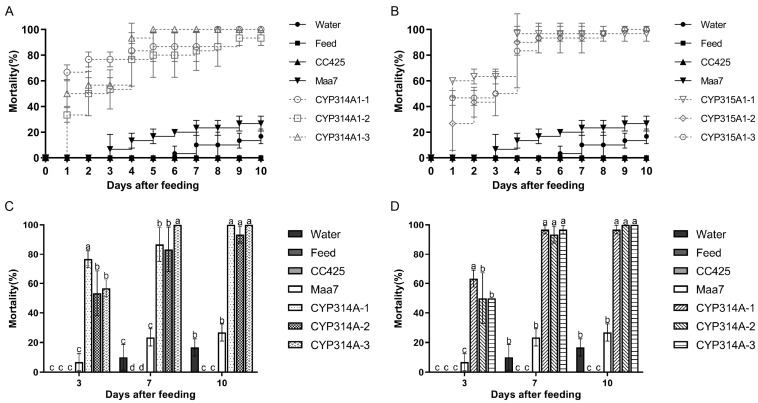
Mortality of *Ae. albopictus* fed with *cyp314a1* and *cyp315a1* RNAi recombinant *Chlamydomonas*. (**A**) Mortality of *Ae. albopictus* fed with *cyp314a1* RNAi recombinant *Chlamydomonas*; (**B**) Mortality of *Ae. albopictus* fed with *cyp315a1* RNAi recombinant *Chlamydomonas*; (**C**) Mortality of *Ae. albopictus* fed with *cyp314a1* RNAi recombinant *Chlamydomonas* on days 3, 7 and 10 after feeding; (**D**) Mortality of *Ae. albopictus* fed with *cyp315a1* RNAi recombinant *Chlamydomonas* on days 3, 7 and 10 after feeding.Water: larvae fed with water; Feed: larvae fed with feed; CC425: larvae fed with *C. reinhardtii CC425*; Maa7: larvae fed with empty plasmid pMaa7IR/XIR transgenic *C. reinhardtii CC425*; CYP314A-1 to CYP314A-3: larvae fed with pMaa7IR/cyp314a1IR transgenic *C. reinhardtii CC425* lines. CYP315A-1 to CYP315A-3: larvae fed with pMaa7 IR/cyp315a1IR transgenic *C. reinhardtii CC425* lines. Each treated or control group contained ten *Aedes* larvae, and the experiments were performed in triplicate for 10 days. Data are expressed as the mean ± SD (*n* = 3), and significant differences (*p* < 0.05, Duncan’s multiple range test) are indicated by different letters.

In the laboratory *Ae. albopictus* larvae feeding assay, larvae fed with the RNAi recombinant *C. reinhardtii* CYP314A(1-3) and CYP315A(1-3) began dying on the second day, with mortality rates of 93.3–100% (95% CI: 88.22–100%) and 96.7–100% (95% CI: 94.11–100%) on the 10th day, respectively. Conversely, no mortality was observed in the larvae fed *C. reinhardtii* CC425 and feed; only 16.7% and 30% of larvae died when fed water and the empty plasmid Maa7IR/XIR transgenic line, respectively. These results suggest that the oral administration of *cyp314a1* and *cyp315a1* RNAi recombinant *C. reinhardtii* is lethal to *Ae. albopictus* larvae (Figure 1).

### 3.3. Effects of RNAi Recombinant Chlorella on Larval Development, and Redox Stress and 20E Compensation Assay

**Figure 2:**
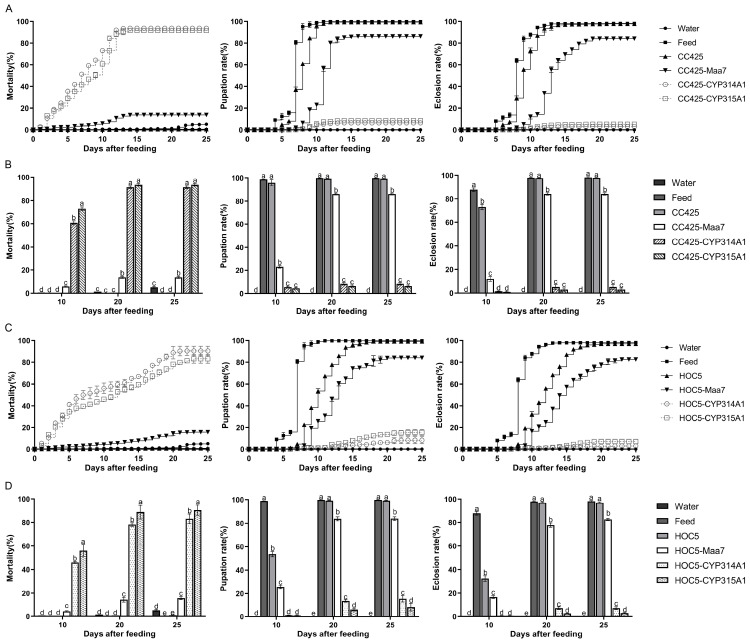
Mortality, pupation, and eclosion rates of *Ae. albopictus* fed with *cyp314a1* and *cyp315a1* RNAi recombinant *Chlamydomonas* (**A**,**B**) and *Chlorella* (**C**,**D**). Water: larvae fed with water; Feed: larvae fed with feed; CC425: larvae fed with *C.reinhardtii CC425*; HOC5: larvae fed with wild *C. vulgaris* HOC5; CC425-Maa7 and HOC5-Maa7: larvae fed with an empty Maa7IR/XIR plasmid in transgenic *Chlorella or Chlamydomonas*; CC425-CYP314A1 and CC425-CYP315A1: larvae fed with *cyp314a1* and *cyp315a1* RNAi recombinant *Chlamydomonas* and *Chlorella* lines. HOC5-CYP314A1 and HOC5-CYP315A1: larvae fed with *cyp314a1* and *cyp315a1* RNAi recombinant *Chlorella* lines. Each treated or control group contained 300 *Aedes* larvae, and the experiments were performed in triplicate over 25 days. Data are expressed as the mean ± SD (*n* = 3), and significant differences (*p* < 0.05, Duncan’s multiple range test) are indicated by different letters.

Approximately 300 L1 *Ae. albopictus* larvae in each treatment group were subjected to a 25-day feeding experiment. The larvae fed with the recombinant *C. reinhardtii* CC425-CYP314A1 and CC425-CYP315A1 began to die on the second day and 93.67% (95% CI: 91.19–96.15%) and 91.56% (95% CI: 88.89–94.22%) died within 25 days, respectively. In the control groups, 5.11% (95% CI: 2.72–7.51%), 0.22% (95% CI: 0–0.69%), 0.67% (95% CI: 0.67–0.67%) and 13.78% (95% CI: 11.67–15.85%) of the larvae died when fed water, feed, *C. reinhardtii* CC425 and transgenic *C. reinhardtii* pMaa7IR/XIR, respectively (Figure 2A). Similarly, the larvae fed recombinant *Chlorella* HOC5-CYP314A1 and HOC5-CYP315A1 began dying on the second day, with 90.44% (95% CI: 79.8–100%) and 83.11% (95% CI: 73.09–93.13%) dying within 25 days, respectively. By contrast, in the control groups, only 5.11% (95% CI: 2.72–7.51%), 0.22% (95% CI: 0–0.69%), 0.78% (95% CI: 0–1.74%) and 15.67% (95% CI: 12.35–18.89%) of larvae died when fed water, feed, *Chlorella* HOC5 and pMaa7IR/XIR transgenic *Chlorella*, respectively (Figure 2B).

Larvae fed the control diet began pupating on day 4, and all had completed pupation by day 12. Those fed *C. reinhardtii* CC425 began pupating on day 5. By day 11, 99.33% (95% CI: 99.33–99.33%) of the larvae had pupated. Larvae fed pMaa7IR/XIR transgenic *C. reinhardtii* CC425 began pupating on day 7, with 86.22% (95% CI: 84.13–88.31%) having pupated by day 16. Larvae fed on recombinant *Chlamydomonas* CC425-CYP314A1 and CC425-CYP315A1 began to pupate on day 6. By day 14, 6.33% (95% CI: 3.85–8.81%) and 8.44% (95% CI: 5.78–11.11%) of the larvae had pupated, respectively (Figure 2A). Similarly, the larvae fed the recombinant *Chlorella* HOC5-CYP314A1 and HOC5-CYP315A1 began to pupate on day 7. By day 25, 8.22% (95% CI: 0.19–16.62%) and 15.44% (95% CI: 8.54–22.34%) of the larvae had pupated, respectively. In the control groups, 0%, 99.78% (95% CI: 99.31–100%), 99.22% (95% CI: 98.26–100%) and 84.00% (95% CI: 80.21–87.8%) of the larvae pupated when fed water, feed, *Chlorella* HOC5 and pMaa7IR/XIR transgenic *Chlorella*, respectively (Figure 2B).

The emergence rates of adult mosquitoes showed that those fed feed began to emerge on day 5, with 98.11% of pupae emerging within 25 days. Those fed *C. reinhardtii* CC425 began to emerge as adults on day 6, with 97.78% (95% CI: 97.31–98.25%) of pupae ultimately emerging. However, Aedes pupae emerged as adults at lower rates of 2.89% (95% CI: 0–6.34%) and 5.11% (95% CI: 0.12–10.1%) when fed recombinant *Chlamydomonas* CC425-CYP314A1 and CC425-CYP315A1 strains, respectively (Figure 2A). Similarly, only 2.89% (95% CI: 1.94–3.84%) and 7.11% (95% CI: 4.21–10.01%) of *Aedes* pupae emerged as adults when fed the recombinant *Chlorella* HOC5-CYP314A1 and HOC5-CYP315A1 strains, respectively. In the control groups, adult mosquito emergence was observed to be 0%, 98.11% (95% CI: 96.38–99.84%), 96.89% (95% CI: 95.16–98.62%) and 82.56% (95% CI: 80.16–84.95%) when the larvae were fed water, feed, *Chlorella* HOC5 or pMaa7IR/XIR transgenic *Chlorella*, respectively (Figure 2B).

**Figure 3:**
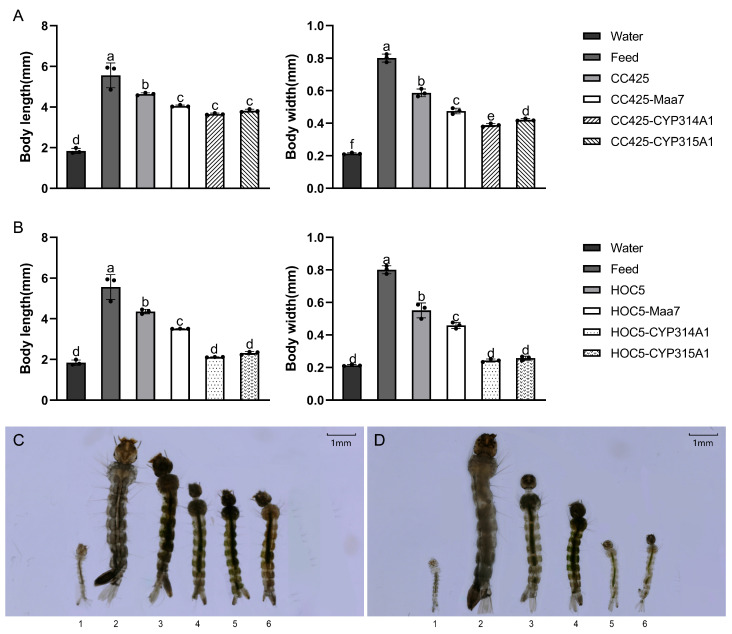
Length and width of larvae. The length and width of L3 larvae from each treatment were measured. (**A**,**C**), larvae fed with recombinant *Chlamydomonas* strains; (**B**,**D**), larvae fed with recombinant *Chlorella* strains. (**C**,**D**), 1, larvae fed water; 2, larvae fed feed; 3, larvae fed *C. reinhardtii* CC425 (**C**) or *C. vulgaris* HOC5 (**D**); 4, larvae fed with empty plasmid Maa7IR/XIR transgenic *Chlamydomonas* (**C**) or *Chlorella* (**D**) strain; 5 and 6, larvae fed with recombinant *Chlamydomonas* (**C**) or *Chlorella* (**D**) strain. Water: water was fed to the larvae; Feed: larvae fed feed; CC425: larvae fed *C. reinhardtii* CC425; HOC5: larvae fed wild *Chlorella vulgaris* HOC5; CC425-Maa7 and HOC5-Maa7: larvae fed with empty plasmid Maa7IR/XIR transgenic *Chlorella* or *Chlamydomonas* strains; CC425-CYP314A1 and CC425-CYP315A1: larvae fed with recombinant *Chlamydomonas* strains. HOC5-CYP314A1 and HOC5-CYP315A1: larvae fed with recombinant *Chlorella* strains. Data are expressed as the mean ± SD (*n* = 3), and significant differences (*p* < 0.05, Duncan’s multiple range test) are indicated by different letters.

The *Ae. albopictus* fed with feed had the longest L3 larval body length (5.56 mm/5.89 mm), followed by mosquitoes fed with *C. reinhardtii* CC425 or *C. vulgaris* HOC5, with body lengths of 4.63 mm and 4.35 mm, respectively. The body length of mosquitoes fed with water was the shortest (1.84 mm), whereas the body length of the mosquitoes fed with recombinant lines was significantly lower (*p* < 0.05) than that of the control group (fed with feed, *C. reinhardtii* CC425 or *C. vulgaris* HOC5) (Figure 3A–D). In terms of body width, the *Ae. albopictus* fed with feed had the widest L3 larval body width (0.80 mm), followed by mosquitoes fed with *C. reinhardtii* CC425 or *C. vulgaris* HOC5, with body widths of 0.59 mm and 0.57 mm, respectively. The body width of mosquitoes fed with water was the narrowest (0.21 mm), whereas the body width of other mosquitoes fed with recombinant strains was significantly lower (*p* < 0.05) than that of the control group (fed with feed, *C. reinhardtii* CC425 or *C. vulgaris* HOC5) (Figure 3A–D). A notable observation was that *Ae. albopictus* fed recombinant *Chlorella* exhibited a reduction in body length and width when compared with *Ae. albopictus* fed recombinant *C. reinhardtii*. (Figure 3A–D).

**Figure 4:**
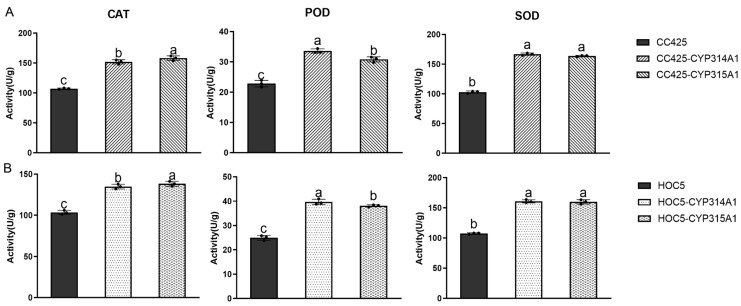
Analysis of CAT, POD and SOD enzyme activities in *Aedes* larvae after feeding on recombinant *Chlamydomonas* (**A**) and *Chlorella* (**B**) strains. CC425: larvae fed with *C. reinhardtii* CC425; HOC5: larvae fed with *C. vulgaris* HOC5; CC425-CYP314A1 and CC425-CYP315A1: larvae fed with recombinant *Chlamydomonas* strains; HOC5-CYP314A1 and HOC5-CYP315A1: larvae fed with recombinant *Chlorella* strains. Data are expressed as the mean ± SD (*n* = 3), and significant differences (*p* < 0.05, Duncan’s multiple range test) are indicated by different letters.

Activity assays of superoxide dismutase (SOD), peroxiredoxin (POD) and catalase (CAT) in *Ae. albopictus* larvae revealed significantly higher enzyme activity in larvae fed *cyp314a1* and *cyp315a1* RNAi recombinant *Chlamydomonas* or *Chlorella* than in the control group (fed *C. reinhardtii* CC425, or *C. vulgaris*) (Figure 4).

**Figure 5:**
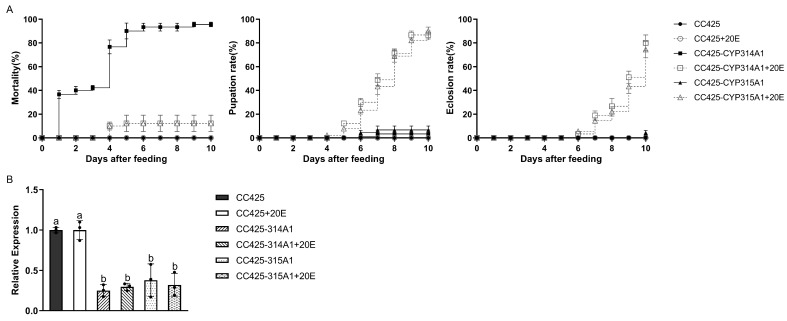
20E compensation test. (**A**) Mortality, pupation, and eclosion rates of *Ae. albopictus* fed with *cyp314a1* and *cyp315a1* RNAi recombinant *Chlamydomonas* with or without 20E; (**B**) The relative *cyp314a1* and *cyp315a1* mRNA levels in L3 larvae fed with recombinant *Chlamydomonas* with or without 20E. CC425: larvae fed *C. reinhardtii* CC425; CC425+20E: larvae fed with C. *reinhardtii* CC425 and 20E; CC425-CYP314A1 and CC425-CYP315A1: larvae fed with recombinant *Chlamydomonas* strains. CC425-CYP314A1+20E and CC425-CYP315A1+20E: larvae fed with the recombinant *Chlamydomonas* strains and 20E. Data are expressed as the mean ±SD (*n* = 3), and significant differences (*p* < 0.05, Duncan’s multiple range test) are indicated by different letters.

Results from the 20E compensation assay revealed that *Ae. albopictus* fed on recombinant *Chlamydomonas* supplemented with 20E exhibited a substantial reduction in larval mortality, as well as a notable increase in pupation and emergence rates (Figure 5A). Expression analysis of *cyp314a1* and *cyp315a1* revealed that mRNA levels of both target genes were significantly reduced (*p* < 0.05) in *Ae. albopictus* fed either *cyp314a1* or *cyp315a1* RNAi recombinant *Chlamydomonas*, regardless of whether 20E was supplemented (Figure 5B).

### 3.4. The Expression of the RNAi Target Genes and the Genes in the Clathrin-Mediated Endocytosis (CME) Pathway

**Figure 6:**
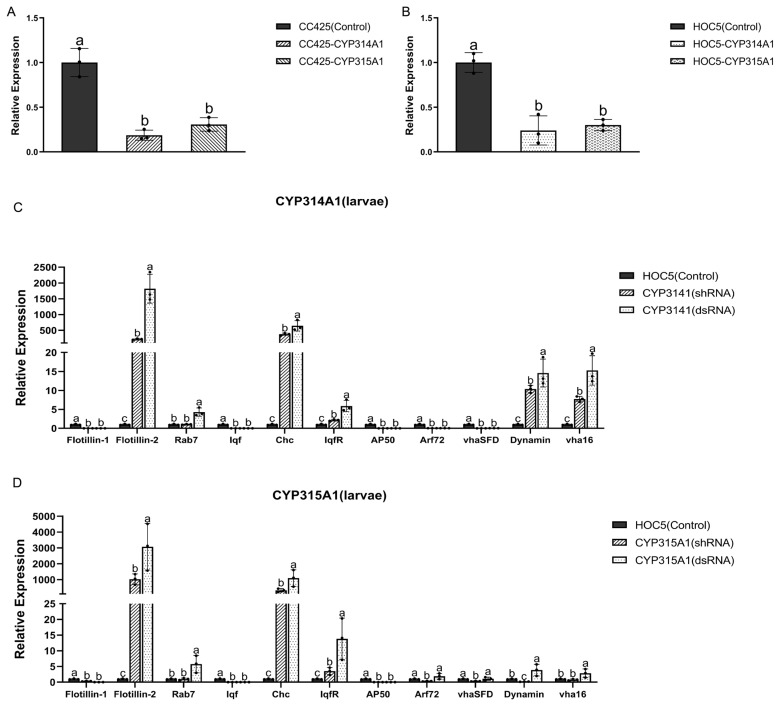
The mRNA levels of genes in *Ae. albopictus* L3 larvae fed with recombinant *Chlamydomonas*/*Chlorella* dsRNA/shRNA. The relative *cyp314a1* and *cyp315a1* mRNA levels in L3 larvae fed with recombinant *Chlamydomonas* (**A**) or *Chlorella* (**B**). The relative *flot-1*, *flot2*, *Rab7*, *iqf*, *Chc*, *iqfR*, *AP50*, *Arf72*, *vhaSFD*, *Dynamin* and *vha16* mRNA levels in *Ae. albopictus* L3 larvae fed with *cyp314a1* dsRNA/shRNA recombinant *Chlorella* (**C**). The relative *flot-1*, *flot2*, *Rab7*, *iqf*, *Chc*, *iqfR*, *AP50*, *Arf72*, *vhaSFD*, *Dynamin* and *vha16* mRNA levels in *Ae. albopictus* L3 larvae fed with *cyp315a1* dsRNA/shRNA recombinant *Chlorella* (**D**). CC425: larvae fed with *C.reinhardtii* CC425; HOC5: larvae fed with wild *C. vulgaris* HOC5; CC425-CYP314A1 and CC425-CYP315A1: larvae fed with recombinant *Chlamydomonas* strains; HOC5-CYP314A1 and HOC5-CYP315A1: larvae fed with recombinant *Chlorella* strains. HOC5-CYP314A1(dsRNA/shRNA) and HOC5-CYP315A1(dsRNA/shRNA): larvae fed with recombinant *Chlorella* containing dsRNA/shRNA. Expression of CME-related genes: Clathrin adapter protein 50 (*AP50*), Clathrin heavy chain (*Chc*), Liquid facets (*lqf*), Liquid facets related protein (*lqfR*), *Dynamin*, Vacuolar H + ATPase 16 kDa (*vha16*), V-type proton ATPase subunit H (*VhaSFD*), Ras-like GTPase (*Rab7*), ADP-ribosylation factor 72 (*Arf72*), Flotillin-1 (*Flot-1*) and Flotillin-2 (*Flot-2*). Results are expressed as the mean ± SE of three independent replicates. For (**A**–**D**), significant differences (*p* < 0.05, Duncan’s multiple range test) are shown by different letters.

Real-time PCR was used to examine *cyp314a1* and *cyp315a1* gene expression in *Ae. albopictus* larvae fed with recombinant *Chlamydomonas* and *Chlorella* strains. Larvae fed with *C. reinhardtii* CC425 or *C. vulgaris* HOC5 were used as controls. The expression levels of the *cyp314a1* and *cyp315a1* in larvae fed the recombinant *Chlamydomonas* or *Chlorella* strains were significantly lower (*p* < 0.05) than in the control larvae (Figure 6A,B). The most substantial decline in gene expression was observed in larvae fed the *cyp314a1* RNAi recombinant *Chlamydomonas* or *Chlorella* strains (82.5% and 80.6% decreases, respectively). These observations collectively indicate that the *cyp314a1* and *cyp315a1* genes in *Ae. albopictus* were effectively silenced by the recombinant *Chlamydomonas* or *Chlorella* strains (Figure 6A,B).

Furthermore, an evaluation was conducted of the levels of several gene transcripts involved in the clathrin-mediated endocytosis (CME) pathway (Supplementary Table S5). These include coat assembly proteins (clathrin adapter AP50 and clathrin heavy chain [Chc]), vesicle formation and budding proteins (liquid facets [lqf], liquid facets-related [lqfR], and dynamin), early endosome proteins (Vha16 and VhaSFD), endosome maturation protein Rab7 and ADP-ribosylation factor-like 1 (Arf72A). Two genes of the flotillin-mediated endocytosis pathway are also included *flot-1* and *flot-2*. The results demonstrated that the mRNA levels of *flot-2*, *Rab7*, *Chc*, *Dynamin* and *vha16* were significantly increased in larvae fed with *cyp314a1* shRNA or recombinant *Chlorella* dsRNA (Figure 6C). Furthermore, the mRNA levels of *flot2*, *Rab7*, *Chc*, *lqfR*, *Dynamin* and *vha16* were also found to be significantly increased in larvae fed with *cyp315a1* shRNA or recombinant *Chlorella* dsRNA (Figure 6D). These results suggested that knockdown of *cyp314a1* or *cyp315a1* influences the expression of CME pathway-related genes more than feeding with ds/shRNA-free *Chlorella* does.

### 3.5. Simulated-Field Trials

**Figure 7:**
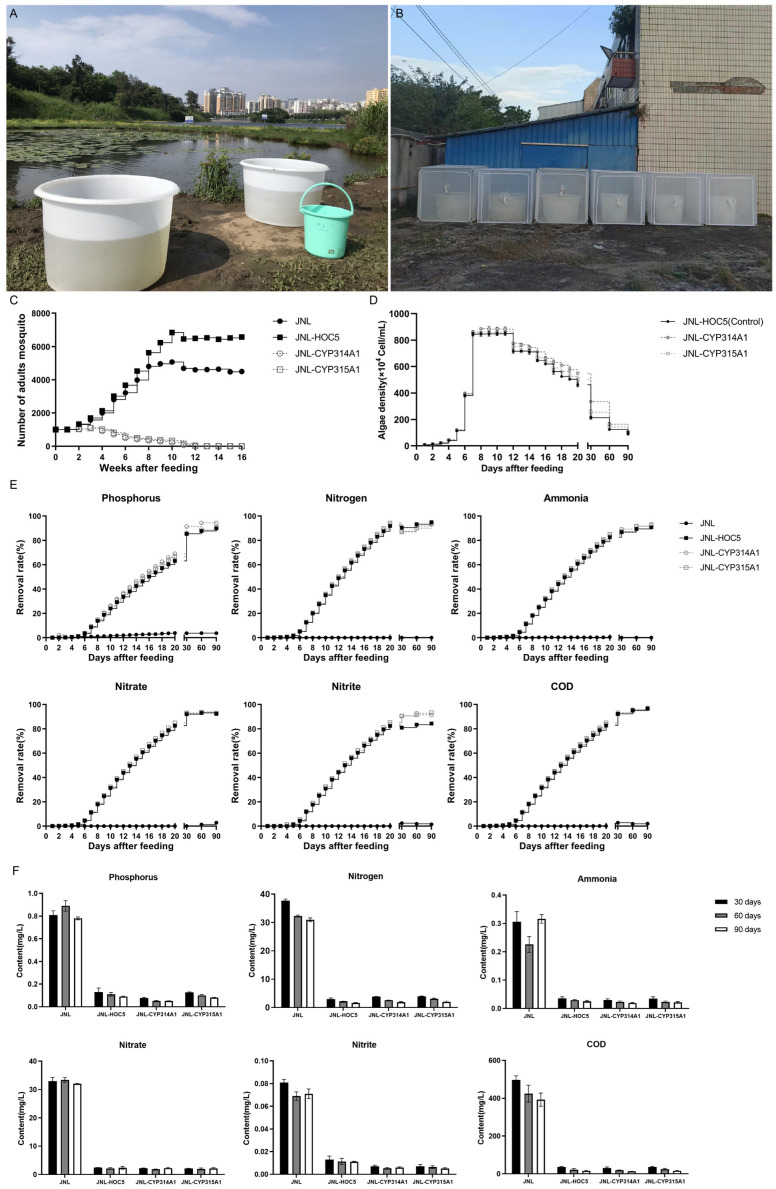
Simulated-field trials in the suburban of Haikou. Test water was taken from Jinniu Ling reservoir (**A**). 200 L of *C. vulgaris* HOC5 were mixed with 600 L of the Jinniu Ling reservoir water in the barrel. Approximately 1000 L1 larvae were placed in each cage, and the number of adult *Ae. albopictus* was counted once a week (**B**). The population and survival rates of *Ae. albopictus* in the JNL (control), JNL-HOC5 (control), JNL-CYP314A1 and JNL-CYP315A1 treatment groups are shown in (**C**). Relative abundance of *Chlorella* in the JNL-HOC5 (control), JNL-CYP314A1 and JNL-CYP315A1 treatment groups (**D**). The removal rates of nitrogen, phosphorus, nitrate, nitrite, ammonia, and COD by RNAi recombinant *Chlorella* (**E**). Nitrogen, phosphorus, nitrate, nitrite, ammonia, and COD content in the JNL-HOC5 (control), JNL-CYP314A1 and JNL-CYP315A1 treatment groups (**F**). JNL: The mosquito lived in water from the Jinniu Ling reservoir alone. JNL-HOC5: The mosquito lived in water from the Jinniu Ling reservoir that was supplemented with wild *Chlorella* HOC5. JNL-CYP314A1 and JNL-CYP315A1: the mosquito lived in water from the Jinniu Ling reservoir that was supplemented with recombinant *Chlorella* HOC5-CYP314A1 and HOC5-CYP315A1.

Large-scale simulated field tests were conducted over a period of 16 weeks. The results demonstrated that the *Ae. albopictus* population fed on Jinniu Ling reservoir water (JNL) increased from 1000 to a maximum of 5063 individuals over 10 weeks. After this, the population slowly decreased to around 4497 individuals after 16 weeks. *Ae. albopictus* raised in Jinniu Ling reservoir water supplemented with wild-type *C. vulgaris* HOC5 (JNL-HOC5) increased to a maximum of 6895 individuals after 10 weeks and then decreased slowly to around 6574 individuals after 16 weeks. In the treatment groups where the water was supplemented with recombinant *Chlorella* HOC5-CYP314A1 or HOC5-CYP315A1, the quantity of *Ae. albopictus* increased in the first three weeks and then decreased from 1100 (JNL-CYP314A1) or 1090 (JNL-CYP315A1) to zero after 13–15 weeks (Figure 7A–C). These results demonstrate the ability of recombinant *Chlorella* HOC5-CYP314A1 and HOC5-CYP315A1 to suppress the *Ae. albopictus* population under simulated field conditions.

To understand the proliferation of RNAi recombinant *Chlorella* in natural water bodies and its effect on water nutrients, a series of water quality tests and *Chlorella* density measurements were conducted on each group of water samples. Following a rapid logarithmic growth phase (days 5–7), the recombinant algal strains entered a stabilisation period (days 8–11), after which they entered a period of reduced growth (days 12–90). The removal rates of nitrogen (N), phosphorus (P), nitrate, nitrite, ammonia and chemical oxygen consumption (COD) from the water were found to be positively correlated with the density of *Chlorella* (Figure 7D). On day 30, the removal rates of N, P, nitrate, nitrite, ammonia, and COD by *C. vulgaris* HOC5, RNAi recombinant *Chlorella* HOC5-CYP314A1 and HOC5-CYP315A1 were 86.96–90.51%, 85.39–91.38%, 91.97–93.03%, 80.88–90.54%, 86.79–89.25% and 92.28–93.32%, respectively (Figure 7E). These values corresponded to water samples containing nitrogen, phosphorus, nitrate, nitrite, ammonia, and COD at concentrations of 2.96–3.95 mg/L, 0.07–0.13 mg/L, 2.14–2.44 mg/L, 0.007–0.013 mg/L, 0.03–0.04 mg/L and 31.08–35.56 mg/L, respectively (Figure 7F). By contrast, the removal rates of N, P, nitrate, nitrite, ammonia, and COD in the control Jinniu Ling Reservoir water samples were only 3.9%, 8.89%, 0.04%, 2.41%, 11.66% and 2.73%, respectively. These levels corresponded to concentrations of 37.71 mg/L, 0.81 mg/L, 32.96 mg/L, 0.08 mg/L, 0.31 mg/L and 496.65 mg/L, respectively. By adding RNAi recombinant *Chlorella*, the levels of N, P, nitrate, nitrite, ammonia and COD in the severely eutrophic Jinniu Ling Reservoir water were reduced to Class V levels (N ≤ 2.0, P ≤ 0.4, nitrate ≤ 10, nitrite ≤ 1, ammonia ≤ 2.0, and COD ≤ 40), in accordance with China’s environmental quality standard for surface water (GB 3838-2002) (Figure 7E,F) [58].

### 3.6. Plankton Abundance and Diversity in Simulated Field Test Waters

To evaluate the impact of RNAi recombinant *Chlorella* on aquatic biological communities, high-throughput analyses of 18S rDNA and 16S rDNA was performed on water samples containing the recombinant *Chlorella* strains HOC5-314A1 and HOC5-315A1. For the 18S rDNA sequencing, 988,356 qualified tags were obtained from the samples after several quality controls steps. After removing unclassified and unique tags from the dataset, the total number of high-quality tags was 818,788. These sequences were then clustered into OTUs with 97% concordance and a total of 1009 OTUs were obtained. Similarly, 1,125,735 qualified tags were obtained from the 16S rDNA sequencing after several quality controls. The total number of high-quality tags was 761,131 and 1213 OTUs were obtained.

**Figure 8:**
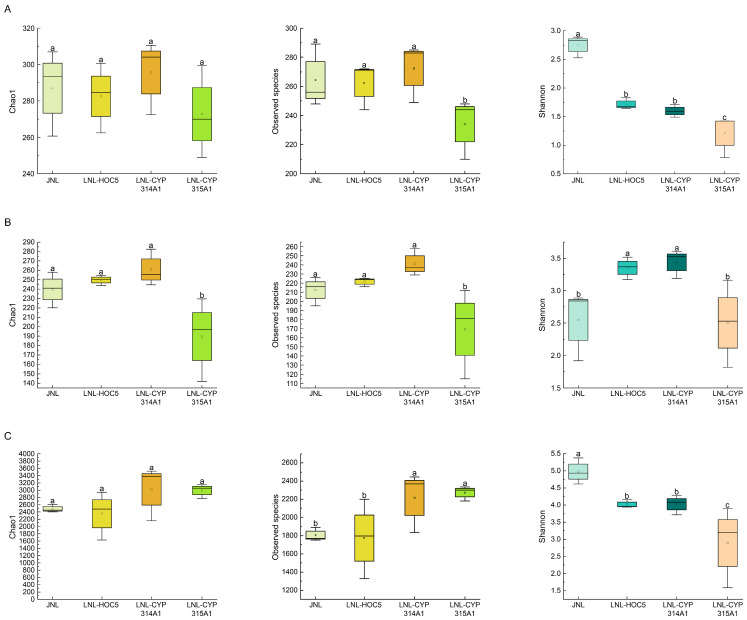
Box plots showing the differences between groups in the Chao1 index, the observed species index and the Shannon index for phytoplankton (**A**), zooplankton (**B**) and prokaryotes (**C**). JNL: water samples from Jinniu Ling Reservoir; JNL-HOC5: water samples from Jinniu Ling Reservoir with wild *Chlorella* HOC5 added; JNL-CYP314A1: water samples from Jinniu Ling Reservoir with RNAi recombinant *Chlorella* HOC5-CYP314A1 added; JNL-CYP315A1: water samples from Jinniu Ling Reservoir with RNAi recombinant *Chlorella* HOC5-CYP315A1 added. Data are expressed as the mean ± SD (*n* = 3), and significant differences (*p* < 0.05, Duncan’s multiple range test) are indicated by different letters.

To assess and compare planktonic biodiversity across samples, we computed richness and diversity indices, including Chao1, observed species and Shannon indices, for phytoplankton, zooplankton, and prokaryote communities. These indices were based on OTUs clustered at 97% sequence similarity, followed by α-diversity analysis (Figure 8). Compared with the control group (JNL group), the phytoplankton Chao1 index and observed species index in the JNL-CYP314A1 treatment group (supplemented with RNAi recombinant *Chlorella* strain HOC5-CYP314A) showed no significant differences. The zooplankton Chao1 index and observed species index exhibited an upward trend but did not reach statistical significance. In contrast, the zooplankton Chao1 index and observed species index in the JNL-CYP315A1 treatment group (supplemented with recombinant strain HOC5-CYP315A1) decreased significantly (*p* < 0.05) compared with the JNL group (Figure 8B). With respect to prokaryotic communities, the JNL-CYP315A1 group displayed a significant increase (*p* < 0.05) in the observed species index relative to the JNL control group (Figure 8C).

Shannon index analysis revealed that, compared with the JNL group, the phytoplankton community diversity in the JNL-CYP314A1 treatment group decreased significantly (*p* < 0.05), whereas zooplankton diversity increased markedly (Figure 8A,B). In addition, both phytoplankton and prokaryotic diversity in the JNL-CYP315A1 group declined significantly (*p* < 0.05) compared with the JNL group (Figure 8A,C). These findings indicate that the supplementation of recombinant *Chlorella* altered the patterns of aquatic biodiversity in the Jinniu Ling Reservoir.

**Figure 9:**
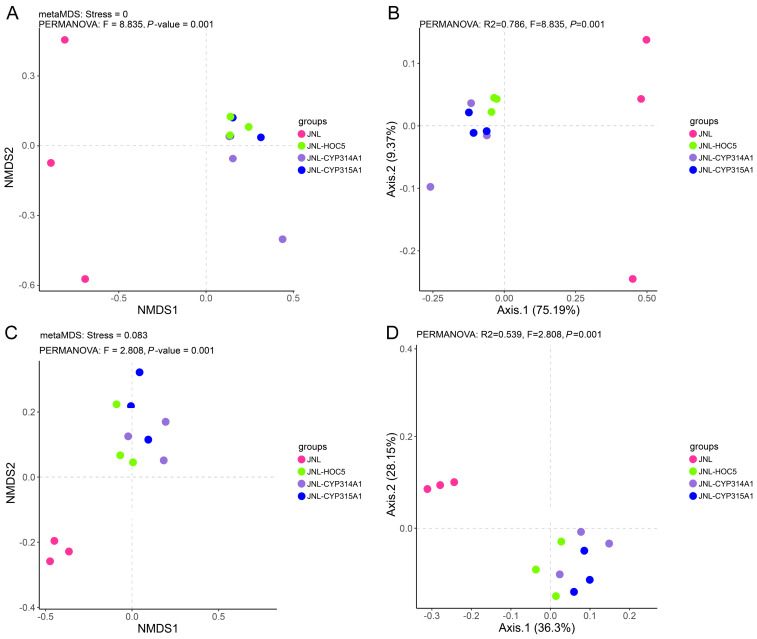
NMDS2 and PCA analyses of eukaryote (**A**,**B**) and prokaryote (**C**,**D**) JNL: water samples from Jinniu Ling Reservoir; JNL-HOC5: water samples from Jinniu Ling Reservoir with wild *Chlorella* HOC5 added; JNL-CYP314A1: water samples from Jinniu Ling Reservoir with RNAi recombinant *Chlorella* HOC5-CYP314A1 added; JNL-CYP315A1: water samples from Jinniu Ling Reservoir with RNAi recombinant *Chlorella* HOC5-CYP315A1 added.

The NMDS and PCA analyses of the eukaryotic communities revealed that JNL (pink points) was clearly separated from the other groups, indicating that the baseline water community structure differed substantially from that of the treatments with added *Chlorella*. JNL-HOC5 (green points) clustered closely with JNL-CYP314A1 (purple points) and JNL-CYP315A1 (blue points), suggesting that the community structure became more similar after the addition of *Chlorella*. However, differences still remained between the RNAi-transgenic *Chlorella* treatments (JNL-CYP314A1, JNL-CYP315A1) and the wild-type HOC5. The PCA results showed that the first principal component (Axis1) explained 75.19% of the variance, indicating that the major community differences were determined by the addition of *Chlorella* and its genotype (Figure 9A,B).

The NMDS and PCA analyses of the prokaryotic communities demonstrated that JNL was distinctly separated from the other groups, indicating that the addition of *Chlorella* had a pronounced effect on the prokaryotic community. JNL-HOC5 overlapped to some extent with JNL-CYP314A1 and JNL-CYP315A1, but the RNAi-treated groups (JNL-CYP314A1, JNL-CYP315A1) displayed a more dispersed distribution, suggesting that the effects of recombinant *Chlorella* on prokaryotic communities were more complex and variable. The PCA results showed that the Axis1 explained 36.3% of the variance and the Axis2 explained 28.15%, indicating that multiple factors contributed to the differences in the prokaryotic community rather than a single dominant factor (Figure 9C,D).

### 3.7. Changes in Plankton in Simulated Field Test Waters

**Figure 10:**
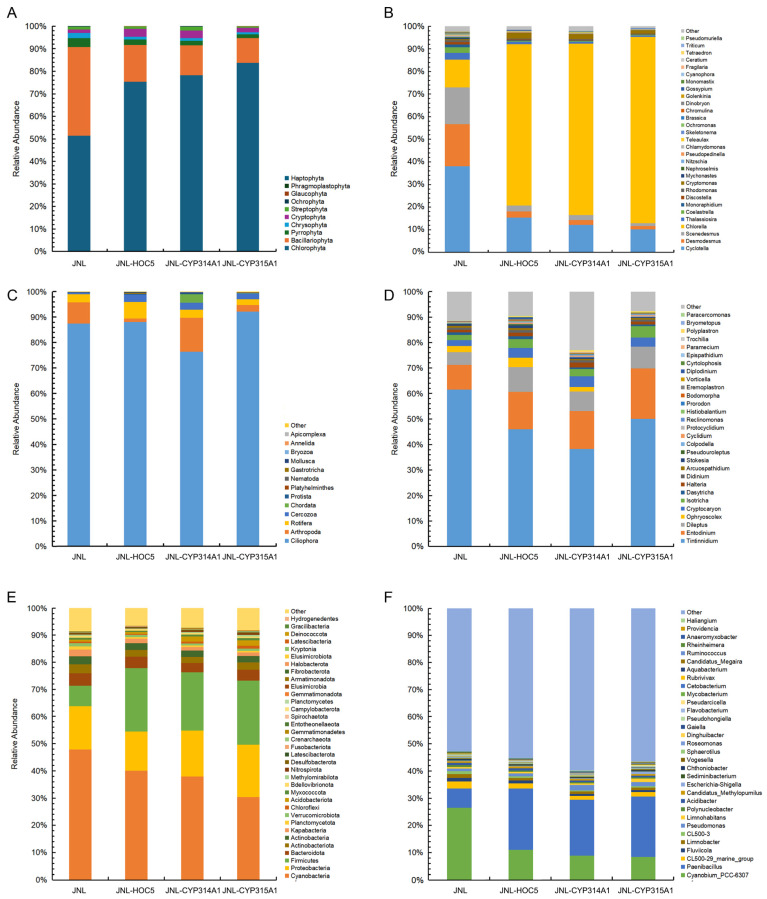
Relative abundance of phytoplankton (**A**,**B**), zooplankton (**C**,**D**) and prokaryotes (**E**,**F**) at the phylum (**A**,**C**,**E**) and genus (**B**,**D**,**F**) levels in simulated field test waters. JNL: water samples from the Jinniu Ling Reservoir; JNL-HOC5: water samples from the Jinniu Ling Reservoir with wild *Chlorella* HOC5 added; JNL-CYP314A1: water samples from the Jinniu Ling Reservoir with RNAi recombinant *Chlorella* HOC5-CYP314A1 added; JNL-CYP315A1: water samples from the Jinniu Ling Reservoir with RNAi recombinant *Chlorella* HOC5-CYP315A1 added. *Chlorella* increased its abundance in the JNL-CYP314A1 and JNL-CYP315A1 treatment groups to 75.80% and 82.41%, respectively, compared to the control JNL group at 12.25% (**B**). *Ciliophora* exhibited abundances of 87.40%, 88.01%, 76.35% and 92.03% in JNL, JNL-HOC5, JNL-CYP314A1 and JNL-CYP315A1 water, respectively (**C**). *Tintinnidium* was the most prevalent species in JNL, JNL-HOC5, JNL-CYP314A1 and JNL-CYP315A1 water, with abundances of 61.56%, 45.87%, 38.28% and 49.99%, respectively (**D**). *Cyanobium_PCC-6307* was the most prevalent species in the JNL, JNL-HOC5, JNL-CYP314A1 and JNL-CYP315A1 water samples, with concentrations of 25.57%, 10.68%, 8.65% and 8.19%, respectively (**F**).

A total of ten groups of eukaryotic microalgae were identified at the phylum level in the Jinniu Ling Reservoir (JNL, control) sample, including *Chlorophyta*, *Chrysophyta*, *Cryptophyta*, *Bacillariophyta*, *Pyrrophyta*, *Streptophyta*, *Ochrophyta*, *Phragmoplastophyta*, *Glaucophyta* and *Haptophyta* (Supplementary Table S6). Of these, 366 OTUs within 83 genera were identified in JNL. *Chlorophyta* exhibited the highest number of OTUs. A total of 120 OTUs were identified within 28 genera of *Chlorophyta*. The second largest class was *Chrysophyta*, comprising 72 OTUs within 15 genera. *Cryptophyta*, *Bacillariophyta* and *Pyrrophyta* also exhibited a relatively high number of OTUs. By contrast, *Phragmoplastophyta*, *Glaucophyta* and *Haptophyta* exhibited lower numbers of OTUs (Supplementary Table S6). In the Jinniu Ling Reservoir water plus *Chlorella* HOC5 group (JNL-HOC5), 356 OTUs belonging to 86 genera were observed. *Chlorophyta* exhibited the highest number of OTUs, with 126 across 27 genera. As shown in Supplementary Table S6, *Chrysophyta* was the second largest phylum, comprising 63 OTUs across 17 genera. In the JNL-CYP314A1 treatment group, 383 OTUs belongs to 92 genera were observed. This was a greater number than in the JNL or JNL-HOC5 control groups. Of these, the number of *Cryptophyta*, *Bacillariophyta* and *Streptophyta* species increased. However, the JNL-CYP315A1 treatment group exhibited a mere 334 OTUs across 80 genera, falling short of the OTUs and genera observed in the control groups (JNL, or JNL-HOC5). Regarding species abundance, *Chlorophyta*, *Bacillariophyta*, *Pyrrophyta*, *Chrysophyta* and *Cryptophyta* were found to be more prevalent in the treatment groups (JNL-CYP314A1 and JNL-CYP315A1) and in the control group (JNL, JNL-HOC5). Compared with the control group, the relative abundance of *Bacillariophyta*, *Pyrrophyta* and *Chrysophyta* decreased in the treatment group, while the relative abundance of *Chlorophyta* increased (Figure 10A).

**Figure 11:**
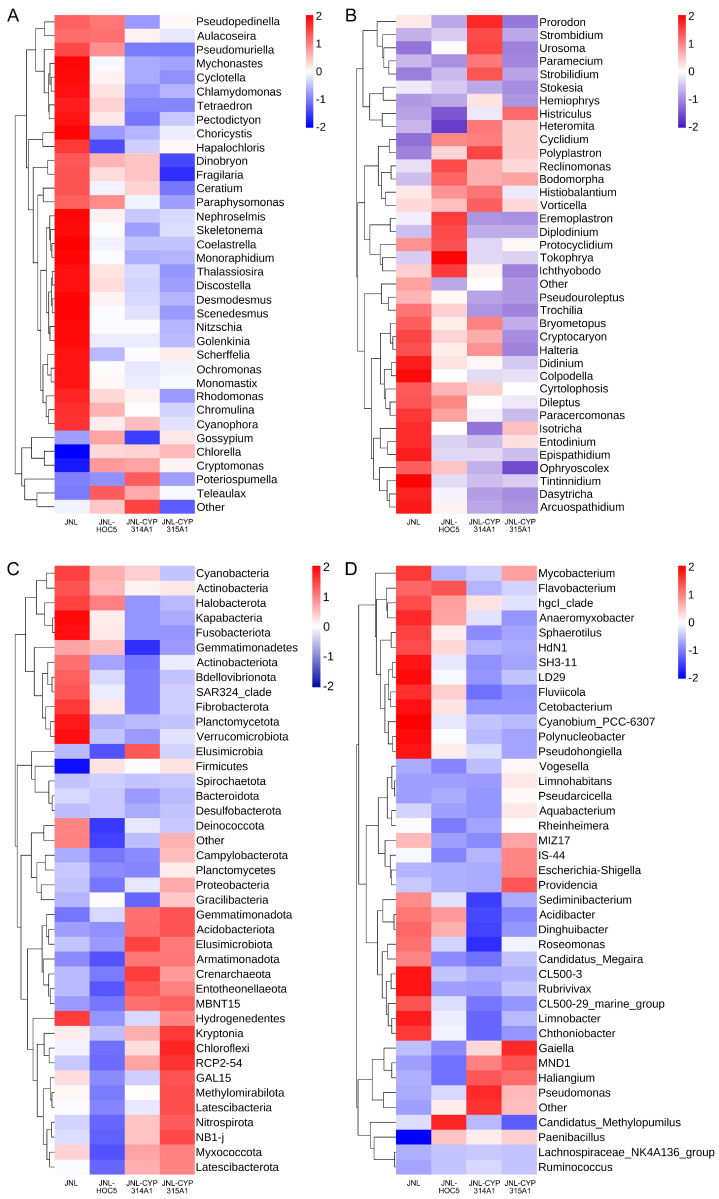
Heat map analysis of phytoplankton at genus level (**A**), zooplankton at genus level (**B**), prokaryotes at phylum level (**C**), and prokaryotic at genus level (**D**) in simulated field test water. JNL: water samples from the Jinniu Ling Reservoir; JNL-HOC5: water samples from the Jinniu Ling Reservoir with wild *Chlorella* HOC5 added; JNL-CYP314A1: water samples from the Jinniu Ling Reservoir with RNAi recombinant *Chlorella* HOC5-CYP314A1 added; JNL-CYP315A1: water samples from the Jinniu Ling Reservoir with RNAi recombinant *Chlorella* HOC5-CYP315A1 added.

Analysis at the Phytoplankton genus level showed that adding recombinant *Chlorella* increased its abundance in the JNL-CYP314A1 and JNL-CYP315A1 treatment groups to 75.80% and 82.41%, respectively, compared to the control JNL group at 12.25%. The abundance of most phytoplankton was decreased in the JNL-CYP314A1 and JNL-CYP315A1 treatment groups, except for *Chlorella*, *Cryptomonas* and *Teleaulax*, whose abundance increased (Figure 10B and Figure 11A). For example, the abundance of *Aulacoseira*, *Cyclotella*, *Fragilaria*, *Skeletonema*, *Thalassiosira*, *Discostella* and *Nitzschia* in the *Bacillariophyta* decreased. The abundance of *Mychonastes*, *Tetraedron*, *Coelastrella*, *Monoraphidium*, *Desmodesmus*, *Scenedesmus* and *Golenkinia* in the *Chlorophyta* also decreased. *Rhodomonas* in the *Cryptophyta*, *Ceratium* in the *Dinophyta*, and *Dinobryon*, *Ochromonas* and *Chromulina* in the *Chrysophyta*, also decreased (Figure 10B and Figure 11A).

Analysis of zooplankton at the phylum level revealed that *Ciliophora*, *Cercozoa*, *Arthropoda* and *Rotifera* occupied the top positions in terms of species number and abundance (Supplementary Table S7 and Figure 10C). Specifically, *Ciliophora* exhibited abundances of 87.40%, 88.01%, 76.35% and 92.03% in JNL, JNL-HOC5, JNL-CYP314A1 and JNL-CYP315A1 water, respectively. Analysis of the normalised zooplankton genus-level data showed that the genera *Tintinnidium*, *Entodinium* and *Dileptus* occupied the top three positions in terms of species abundance. *Tintinnidium* was the most prevalent species in JNL, JNL-HOC5, JNL-CYP314A1 and JNL-CYP315A1 water, with abundances of 61.56%, 45.87%, 38.28% and 49.99%, respectively (Figure 10C).

Analysis of differences in species abundance revealed higher abundances of *Cilicophora* (*Histriculus*, *Cyclidium*, *Polyplastron*, *Histiobalantium* and *Vorticella*) and *Protozoa* (*Heteromita*, *Reclinomonas* and *Bodomorpha*) in treatment groups JNL-CYP314A1 and JNL-CYP315A1 than in the control group, JNL. In contrast, the *Cilicophora* (*Pseudouroleptus*, *Trochilia*, *Bryometopus*, *Cryptocaryon*, *Halteria*, *Didinium*, *Dileptus*, *Paracercomonas* and *Epispathidium*), and rumen-specialised groups of ruminants (*Entodinium*, *Ophryoscolex* and *Dasytricha*), as well as other protists such as *Colpodella*, *Cytolphosis*, *Arcuospathidium* and *Tintinnidium* (which consume diatoms and dinoflagellates), were reduced in abundance (Figure 10C,D and Figure 11B).

### 3.8. Changes in Prokaryotes in Simulated Field Test Waters

Analysis of the water samples using 16S rDNA high-throughput sequencing revealed the presence of high concentrations of *Cyanobacteria*, *Proteobacteria*, *Firmicutes*, *Bacteroidota*, *Actinobacteriota* and *Actinobacteria* in the control (JNL and JNL-HOC5) and treatment (JNL-CYP314A1 and JNL-CYP315A1) groups (Figure 10E). *Cyanobacteria* were the most prevalent of these, with proportions of 47.57%, 39.96%, 37.78% and 30.10% in JNL, JNL-HOC5, JNL-CYP314A1 and JNL-CYP315A1, respectively. At the genus level, the top three positions in terms of abundance were occupied by *Cyanobium_PCC-6307*, *Paenibacillus*, and *CL500-29_marine_group*. As shown in Figure 10F, *Cyanobium_PCC-6307* was the most prevalent species in the JNL, JNL-HOC5, JNL-CYP314A1 and JNL-CYP315A1 water samples, with concentrations of 25.57%, 10.68%, 8.65% and 8.19%, respectively.

*Cyanobium_PCC-6307* belongs to the family *Cyanobiaceae*, a group of photosynthetic, autotrophic prokaryotes that are widely distributed in freshwater, marine and wetland environments. It has a thylakoid structure typical of cyanobacteria and fixes carbon through photosynthesis, while also being involved in nitrogen metabolism. It is one of the dominant bacterial genera in natural or artificial waters and is an indicator species of eutrophication [68,69,70,71]. The abundance of *Cyanobium_PCC-6307* in the JNL-CYP314A1 and JNL-CYP315A1 waters decreased as the N and P contents decreased. *Paenibacillus* is a genus of Gram-positive bacteria widely distributed in the natural environment. It belongs to the phylum *Firmicutes*, the class Bacilli, and the family *Paenibacillaceae*. The genus currently comprises over 150 species, around 20 of which have the ability to fix nitrogen, secrete organic acids to dissolve insoluble phosphorus in water, degrade organic pollutants and reduce the eutrophication of water [72,73,74,75]. The results showed that the *Paenibacillus* abundance in treated JNL-CYP314A1 and JNL-CYP315A1 water increased significantly (19.92% and 21.54%, respectively) compared to the control (JNL, 6.79%) (Figure 10F). The *CL500-29_marine_group* belongs to the phylum *Actinobacteriota*, which contains the dominant bacteria in freshwater and offshore environments. The *CL500-29_marine_group* is an important phylum of *Actinobacteria* with significant ecological functions. It can efficiently utilise a variety of carbon sources, such as amino acids, carboxylic acids and lipids. Its abundance is positively correlated with lake carbon concentration and nitrogen and phosphorus removal efficiency, suggesting that it is closely related to water purification capacity [76,77,78,79,80]. The sequencing results obtained in this study align with the following observations: the introduction of recombinant *Chlorella* into the treatment group let to lower concentrations of carbon, nitrogen and phosphorus in the water, as well as a decrease in the abundance of the CL500-29_marine_group.

Heat map-based analysis of differences in the abundance of prokaryotic species at the phylum level after normalisation revealed that the abundances of *Gemmatimonadota*, *Acidobacteriota*, *Elusimicrobiota*, *Armatimonadota*, *Crenarchaeota*, *Entotheonellaeota*, *Kryptonia*, *Chloroflexi*, *Methylomirabilota*, *Latescibacteria*, *Nitrospirota*, *Myxococcota* and *Latescibacterota* were higher in the treatment groups (JNL-CYP314A1 and JNL-CYP315A1) than in the control group (JNL). Conversely, the abundances of *Cyanobacteria*, *Actinobacteria*, *Halobacterota*, *Kapabacteria*, *Fusobacteriota*, *Gemmatimonadetes*, *Actinobacteriota*, *Bdellovibrionota*, *Fibrobacterota*, *Planctomycetota*, *Verrucomicrobiota*, and *Deinococcota* were markedly lower in the treatment groups than in the control group (Figure 10E and Figure 11C). At the genus level, the analysis revealed that the abundances of *Vogesella*, *Limnohabitans*, *Pseudarcicella*, *Aquabacterium*, *Rheinheimera*, *Escherichia-Shigella*, *Providencia*, *Gaiella*, *Haliangium*, *Pseudomonas*, *Candidatus*, *Methylopumilus*, and *Paenibacillus* were higher in the treatment groups (JNL-CYP314A1 and JNL-CYP315A1) than in the control group (JNL). By contrast, the abundance of the following bacteria decreased in the treatment groups compared to the control group: *Mycobacterium*, *Flavobacterium*, *Anaeromyxobacter*, *Sphaerotilus*, *Fluviicola*, *Cetobacterium*, *Cyanobium_PCC-6307*, *Polynucleobacter*, *Pseudohongiella*, *Sediminibacterium*, *Acidibacter*, *Dinghuibacter*, *Roseomonas*, *Rubrivivax*. CL500-29_marine_group, *Limnobacter* and *Chthoniobacter* were lower in the treatment groups than in the control group (Figure 10F and Figure 11D).

## 4. Discussion

*Ae. albopictus* is recognised as a vector of significant public health vector, primarily transmitting viruses such as the Dengue, Yellow fever, Chikungunya and Zika viruses. These diseases cause substantial human mortality each year [81]. In the absence of effective therapies or vaccines, control the vectors remains the most effective strategy for preventing the spread of these pathogens. Extensive research has demonstrated that RNAi-based biopesticides represent a new generation of pesticides and are a powerful tool for controlling insect populations. Compared to other strategies, RNAi biopesticides are more environmentally friendly. However, they also face challenges related to target-site resistance, as evidenced by the emergence of resistance issues following the widespread adoption of transgenic insect-resistant cotton [82,83,84,85]. While the high efficiency and specificity of RNAi biopesticides have garnered significant attention, the environmental impacts of their large-scale deployment and application have not yet been comprehensively evaluated [86,87].

In this study, the *cyp314a1* and *cyp315a1* genes of *Ae. albopictus* were selected as RNAi targets. CYP450 is an ancient and multifunctional superfamily of monooxygenases [22]. Insects possess four CYP450 clans with roles in metabolic detoxification, growth regulation and development [25,26]. CYP450 enzymes are involved in ecdysone biosynthesis. Specifically, shadow (*sad*, CYP315A1) and shade (*shd*, CYP314A1) catalyse the final steps of cholesterol conversion into the biologically active 20-hydroxyecdysone (20E) [88,89,90]. As they are critical to ecdysone synthesis, the *cyp314a1* and *cyp315a1* genes are predominantly found in arthropods, suggesting they have minimal impact on humans, higher animals and plants. Our analysis of *Ae. albopictus* CYP450 genes revealed that the species harbours 81 such genes, distributed across Clan 2 (9 genes), Clan 3 (52 genes), Clan 4 (27 genes) and MITO (7 genes). Genes involved in ecdysone synthesis belong to Clan 2 (e.g., *cyp306a1*, *cyp307a1* and *cyp18a1*) and the MITO family (e.g., *cyp302a1*, *cyp314a1* and *cyp315a1*) (Supplementary Figure S5).

Observations demonstrated that *Ae. albopictus* larvae fed with *cyp314a1* or *cyp315a1* recombinant RNAi *Chlamydomonas* exhibited mortality rates of 93.3–100% or 96.7–100%, respectively, within a 10-day period (Figure 1). In a feeding trial involving 300 mosquitoes, larvae fed *cyp314a1* or *cyp315a1* recombinant RNAi *Chlamydomonas* showed mortality rates of 93.67% and 91.56% over 25 days, respectively. Those fed *cyp314a1* or *cyp315a1* recombinant RNAi *Chlorella* exhibited mortality rates of 90.44% and 83.11%, respectively (Figure 2). These results confirm that the oral administration of *cyp314a1* or *cyp315a1* RNAi *Chlamydomonas/Chlorella* has a lethal effect on *Ae. albopictus* larvae. Pupation rates were 6.33% and 8.44% for larvae fed with *cyp314a1* or *cyp315a1* RNAi recombinant *Chlamydomonas*, respectively, and 8.22% and 15.44% for larvae fed with *cyp314a1* or *cyp315a1* RNAi recombinant *Chlorella*, respectively (Figure 2). With regard to adult emergence, the emergence rates of larvae fed with *cyp314a1* or *cyp315a1* RNAi recombinant *Chlamydomonas* were 2.89% and 5.11%, respectively, while the emergence rates of those fed with *cyp314a1* or *cyp315a1* RNAi recombinant *Chlorella* were 2.89% and 7.11%, respectively (Figure 2). These findings suggest that recombinant algal strains expressing *Cyp314a1* or *Cyp315a1* RNAi significantly disrupt the pupation and adult emergence processes of *Ae. albopictus*.

Using *Ae. albopictus* larvae as a model, we observed that feeding them *cyp314a1* or *cyp315a1* RNAi recombinant algal strains significantly increased the enzymatic activities of SOD, POD and CAT compared to control groups (fed *C. reinhardtii* CC425 or *C. vulgaris* HOC5), indicating an enhanced redox stress (Figure 4). The 20E rescue assay revealed that supplementing *cyp314a1* or *cyp315a1* RNAi recombinant *Chlamydomonas* with exogenous 20E substantially reduced larval mortality and restored pupation and adult emergence rates (Figure 5A). 20E regulates processes such as moulting, metamorphosis, and tissue apoptosis, and is the key hormone required for maintaining the transition from larva to adult. Deficiency or reduction of 20E leads to developmental arrest and moulting failure in larvae. Redox stress induces cellular damage, mitochondrial disfunction, and energy metabolism disorders. The combined effects of these two factors ultimately result in larval death.

Studies have demonstrated that the dsRNA and shRNA of *cyp314a1* or *cyp315a1* likely penetrate the interior of mosquitoes via the clathrin-mediated endocytosis (CME) pathway in intestinal epithelial cells. In contrast, invertebrates such as nematodes use the SID pathway to transport dsRNA into and between cells [91]. However, insects primarily absorb exogenous dsRNA through CME [92,93]. Following the knockout of the *cyp314a1* or *cyp315a1* gene, the mRNA levels of certain key CME pathway genes increased (see Figure 6C,D), corroborating the findings of Abbasi et al. (2020) regarding *Ae. aegypti* [94].

Subsequent simulated-field trials revealed that RNAi-recombinant *Chlorella* reduced the *Ae. albopictus* population from 1000 to zero within 16 weeks (see Figure 7A–C). It also reduced the levels of nitrogen, phosphorus, ammonia, nitrates, nitrites and COD in the experimental water (Jinniu Ling Reservoir), upgrading it to China’s Class V surface water standard (GB 3838-2002) (see Figure 7E,F) [58].

To understand the effect of RNAi recombinant *Chlorella* on the test water’s biological populations, 18S and 16S rDNA high-throughput sequencing analyses were performed. The results showed that the rapid reproduction of the recombinant *Chlorella* caused a bloom that consumed nutrients such as C, N and P at a rapid rate. Competition for nutrient salts directly inhibits the growth of other algae, such as diatoms (e.g., *Aulacoseira*, *Cyclotella* and *Thalassiosira)* and green algae (e.g., *Scenedesmus* and *Coelastrella*) [95,96,97]. Competition for light resources results in decreased light transmittance (increased turbidity), leading to the decline of photosynthesis-dependent non-planktonic algae (e.g., the attached diatom *Fragilaria* and the green algae *Desmodesmus*, *Tetraedron* and *Monoraphidium*) due to insufficient light [68]. However, motile species such as *Cryptomonas* and *Teleaulax* can acquire light energy through vertical migration, giving them a competitive advantage (see Figure 10A,B and Figure 11A) [98].

During the algal bloom triggered by recombinant RNAi in *Chlorella*, the water body’s dissolved oxygen (DO) levels exhibited significant diurnal fluctuations: photosynthesis resulted in DO supersaturation during the day, whereas respiration caused hypoxic stress at night. These extreme redox oscillations periodically stressed aerobic zooplankton (*Didinium* and *Halteria*) physiologically, driving the dynamic remodelling of the microbial community structure. The drastic decrease in DO at night significantly inhibited the proliferation of the aerobic groups *Cyanobium PCC-6307*, *Limnobacter*, *Chthoniobacter* and *Actinobacteria*. Meanwhile, the abundance of facultative and specialised anaerobic microorganisms such as *Methylomirabilota* and *Nitrospirota* increased. The biomass of the strict anaerobe *Chloroflexi* and *Elusimicrobiota* increased significantly compared to the control due to the enhanced anaerobic microenvironment in the bottom sediment (Figure 11C) [99,100,101]. DOM and POM, produced by the decomposition of *Chlorella* residues during the decline of the algal bloom, provided preferential carbon sources for heterotrophic flora, including *Pseudarcicella*, *Aquabacterium*, *Vogesella*, *Rheinheimera*, *Pseudomonas*, *Paenibacillus* and *Haliangium*, as well as the *Myxococcota*. The release of nitrogen and phosphorus derived from the algae, activates the metabolic activities of the nitrifying bacterium *Crenarchaeota*, the denitrifying bacterium *Nitrospirota* and the sulfur-oxidising bacterium *Gemmatimonadota*. Conversely, *Cyanobacteria*, *Planctomycetes* and *Halobacteria* decreased in abundance due to inferior competition for carbon and nitrogen (Figure 10E,F and Figure 11C,D) [102,103,104,105,106].

Analysis based on alpha diversity data showed that adding the wild type of *C. vulgaris* HOC5 slightly altered the biotope but did not significantly reduce diversity (similar Chao1 and Shannon indices) or impact community stability overall. However, the alpha diversity (e.g., observed species and Shannon index) of the JNL-CYP315A treatment group differed significantly from that of the control JNL group. This indicates that the RNAi recombinant algal strain significantly altered the structure of the biological community, resulting in fewer zooplankton species and decreased diversity (see Figure 8). The significant difference in observed species and Shannon index between the JNL-CYP314A1 and JNL-CYP315A1 treatment groups suggests that the difference in biological impact on the surrounding environment may be due to variations between the CYP314A1 and CYP315A1 RNAi recombinant algal strains. Analysis of the dsRNA target fragments revealed multiple differential fragments between the two strains. BLAST comparison of fragments belonging to the CYP315A1 dsRNA target region in the NCBI database showed that multiple fragments were homologous (≥80%) with some of the less abundant zooplankton species. This may explain the reduction in zooplankton species in the JNL-CYP315A treatment group (Figure 8B and Supplementary Table S8).

In conclusion, changes in environmental water bodies are mainly due to the release of RNAi recombinant *Chlorella* and community reconfiguration due to resource competition (e.g., nitrogen–phosphorus competition between *Chlorella* and diatoms), which is induced by algal blooms. Other factors include anaerobic bacterial proliferation and aerobic bacterial decline caused by anaerobic conditions.

While this study has made some progress, the impact of exogenous DNA fragments (particularly antibiotic resistance genes) carried by RNAi recombinant *Chlorella* on environmental organisms (including those in the direct and indirect food chains) requires further evaluation. Lake algal blooms generally go through a cycle of ‘recovery-emergence-decay-dormancy’ [107]. During the bloom stage, the productivity of phytoplankton increases significantly [108], whereas during the decay and decomposition stage, the apoptosis of algal cells releases large quantities of sugars, proteins, and lignin into the water. These substances then become an important source of DOM in the water [109]. However, this study only examined the biotope composition and structure during the RNAi recombinant *Chlorella* bloom, and did not consider what happened after the bloom ended. Future studies will explore this to clarify the environmental impact of RNAi recombinant *Chlorella* algal blooms. Current evidence suggests that the environmental impact of such blooms is limited and similar to that of green algal blooms in urban eutrophic lakes. As the bloom enters the decay and dormancy stage, the biological populations in the water are restructured as nutrients are depleted, which increases species diversity and restores the population balance.

## 5. Conclusions

In this study, we selected the *cyp314a1* and *cyp315a1*, two genes involved in the ecdysone biosynthesis pathway of *Ae. albopictus*, as targets for RNA interference (RNAi). The recombinant *Chlamydomonas*/*Chlorella* mosquito biocide obtained through genetic transformation exhibited high lethality towards *Ae. albopictus* larvae. Simulated field trials of the recombinant *Chlorella* biocide effectively controlled the *Ae. albopictus* population. The recombinant *Chlorella* induced a bloom, altering the population structure of the water and inhibiting the growth of most phytoplankton through trophic competition. The anaerobic environment created by the bloom subsequently triggered the proliferation of anaerobic bacteria and the decline of aerobic bacteria. This study is an important addition to research on the use of recombinant microalgae as mosquito biocides.

## Data Availability

All relevant data are within the manuscript and its Supporting Information Files.

## Supplementary Materials

The following supporting information can be downloaded at https://www.mdpi.com/article/10.3390/insects16101033/s1. Table S1: Primers used in this study; Table S2: Origin, taxonomy and GenBank accession number of the CYP314A1 orthologues used in the present study; Table S3: Origin, taxonomy and GenBank accession number of the CYP315A1 orthologues used in this study; Table S4: *Ae. albopictus* CYP450 information; Table S5: *Ae. albopictus* endocytic genes and related sequences; Table S6: The number of genera and OTUs in different groups of phytoplankton at the phylum level; Table S7: The number of genera and OTUs in different groups of zooplankton at the phylum level; Table S8: Comparison of sequence homology between *Ae. albopictus cyp315a1* dsRNA region and zooplankton target genes; Figure S1: Phylogenetic relationship of CYP314A1 and analysis of their conserved domains; Figure S2: Phylogenetic relationship of CYP315A1 and analysis of their conserved domains; Figure S3: The alignment of the amino acid sequences of CYP314A1 in *Ae. albopictus* and its orthologues in the *Arthropoda*; Figure S4: The alignment of the amino acid sequences of CYP315A1 in *Ae. albopictus* and its orthologues in the *Arthropoda*; Figure S5: Phylogeny of *Ae. albopictus* cytochrome P450; Figure S6: PCR electrophoresis results of recombinant plasmid pMaa7IR/cyp314a1IR(A) and pMaa7IR/cyp315a1IR (B) transgenic algal lines; Figure S7: Sequence of *cyp314a1* and *cyp315a1* shRNA and predicted shRNA secondary structure.
