# Supplementary material for: Silencing the cyp314a1 and cyp315a1 Genes in the Aedes albopictus 20E Synthetic Pathway for Mosquito Control and Assessing Algal Blooms Induced by Recombinant RNAi Microalgae
Source: Insects. 2025 Oct 7;16(10):1033. doi: 10.3390/insects16101033 (PMC12564949; doi:10.3390/insects16101033)
Supplement: Supplementary file 1 [file insects-16-01033-s001.zip › insects-3860222-supplementary.pdf]

Article

Silencing the *cyp314a1* and *cyp315a1* Genes in the *Aedes albopictus* 20E Synthetic Pathway for Mosquito Control and Assessing Algal Blooms Induced by Recombinant RNAi *Microalgae*

Xiaodong Deng [1,2,3], Changhao He [4], Chunmei Xue [5], Dianlong Xu [1], Juncai Li [4] and Xiaowen Fei [4,*]

1 Institute of Tropical Bioscience and Biotechnology, Chinese Academy of Tropical Agricultural Science & Key Laboratory of Biology and Genetic Resources of Tropical Crops of Hainan Province, Hainan Institute for Tropical Agricultural Resources, Haikou 571101, China
2 Hainan Provincial Key Laboratory for Functional Components Research and Utilization of Marine Bio-Resources, Haikou 571101, China
3 Zhanjiang Experimental Station, CATAS, Zhanjiang 524013, China
4 Department of Biochemistry and Molecular Biology & Key Laboratory of Tropical Translational Medicine of Ministry of Education, College of Basic Medical Sciences, Hainan Medical University, Haikou 571199, China
5 College of Life Sciences and Agriculture, Jiamusi University, Jiamusi 154007, China
* Correspondence: xiaowen.fei@muhn.edu.cn

Table S1. Primers used in this study.

Genes	Primer name	Sequence (5'→3')	Gene ID (Vectorbase /Gene bank)
cyp314a1	314A-F	GGTGCTGAAATATCCTAGCAAG	AALFPA_063667
	314A-R	GACTGATGTACATGAAGGTCG	
cyp315a1	315A-F	GCTCCGCAAGCAATATACTG	AALFPA_079547
	315A-R	GTAATAAGTCCAGACCTGTGGC	
AP50	*AP50-F*	ATCAAGTATCGCCGCAAT	AALC636_033072
	AP50-R	ACGCACCTCAATCTTCTG	
Chc	*Chc-F*	AGCAGTGGATGTCTTCTTC	AALC636_022815
	Chc-R	GCATTAGCAGCAACCTTG	
lqf	*lqf-F*	CAGTGAAGAGTATGGCAGAT	AALC636_033617
	lqf-R	AGAAGCAGGAATGGAGTTG	
lqfR	lqfR-F	CAGTGAAGAGTATGGCAGAT	AALFPA_060726
	lqfR-R	AGAAGCAGGAATGGAGTTG	
Dynamin	*Dynamin-F*	TCAATCAGCAACTCACCAA	AALFPA_067314
	Dynamin-R	TGTATTCCTCCACCTCCTT	
Vha16	*Vha16-F*	GCGTTAGGAGCAGCATAT	AALC636_000071
	Vha16-R	TGTATTCCTCCACCTCCTT	
VhaSFD	*VhaSFD-F*	GAACCTTGAGCAGCAGAA	AALFPA_048766
	VhaSFD-R	ATCATCAGACACTTCCAACA	
Rab7	*Rab7-F*	GGAAGACCTCGTTGATGAA	AALC636_020391
	Rab7-R	CTGTCGTCCACCATTACTT	
Arf72	*Arf72-F*	CAGACGCAGTCATCTATGT	AALFPA_076650
	Arf72-R	ATCCACCAACCACTCCAT	
Flot-1	*Flot-1-F*	TTGTCTTGCCGTGTATTCA	AALFPA_080924
	Flot-1-R	CTCCAGTGTCTCCAATGC	
Flot-2	*Flot-2-F*	GGTGAAGGTGATGACTGAA	AALC636_000218
	Flot-2-R	GTTAGCGTGCCAAGGATA	
rps17	*rps17*-F	AAGAAGTGGCCATCATTCCA	AAEL004175
	rps17-R	GGTCTCCGGGTCGACTTC	
pCAMBIA1302	*1302-F*	TTACCCAACTTAATCGCCTTGCAG	AF234298
	1302-R	TATCGCAATGATGGCATTTGTAGG	
Maa7IR/XIR	*Maa7-F*	ATCGATACCGTCGACCTCGAG	AY710294.1
	Maa7-R	CGCGGTGAGCCACCGGAACGG	
16S-rDNA	*16SV34-F*	CCTAYGGGRBGCASCAG	
	16SV34-R	GGACTACNNGGGTATCTAAT	
18S-rDNA	*D514-F*	TCCAGCTCCAATAGCGTA	
	B706-R	AATCCRAGAATTTCACCTCT	

Table S2. Origin, taxonomy and GenBank accession number of the CYP314A1 orthologues used in the present study.

Species	Class	Order	Gene ID
Aedes albopictus	Insecta	Diptera	XM_062855588.1
Aedes aegypti	Insecta	Diptera	XM_021856596.1
Culex quinquefasciatus	Insecta	Diptera	XM_038263889.1
Anopheles cruzii	Insecta	Diptera	XM_053009316.1
Ctenocephalides felis	Insecta	Diptera	XM_026609440.1
Drosophila melanogaster	Insecta	Diptera	AF484414.1
Nilaparvata lugens	Insecta	Hemiptera	KM216996.1
Sogatella furcifera	Insecta	Hemiptera	KX660711.1
Pediculus humanus corporis	Insecta	Phthiraptera	XM_002425597.1
Cryptotermes secundus	Insecta	Blattodea	XM_033752786.1
Periplaneta americana	Insecta	Blattodea	XM_069829562.1
Anabrus simplex	Insecta	Orthoptera	XM_067142949.2
Schistocerca cancellata	Insecta	Orthoptera	XM_049925012.1
Athalia rosae	Insecta	Hymenoptera	XM_012412319.3
Neodiprion pinetum	Insecta	Hymenoptera	XM_046624303.2
Colaphellus bowringi	Insecta	Coleoptera	OK032508.1
Pagiophloeus tsushimanus	Insecta	Coleoptera	PP098266.1
Spodoptera frugiperda	Insecta	Lepidoptera	MN480678.1
Pardosa pseudoannulata	Arachnida	Araneae	MN782369.1
Polyphagotarsonemus latus	Arachnida	Acarina	OR456302.1
Chamberlinius hualienensis	Diplopoda	Polydesmida	LC125380.1
Daphnia sinensis	Crustacea	Cladocera	WJBH02000003.1
Diaphanosoma celebensis	Crustacea	Cladocera	MK336427.1
Lepeophtheirus salmonis	Crustacea	Siphonostomatoida	MN551218.1
Tigriopus japonicus	Crustacea	Harpacticoida	KF640018.1

Table S3. Origin, taxonomy and GenBank accession number of the CYP315A1 orthologues used in this study.

Species	Class	Order	Gene ID
Aedes albopictus	Insecta	Diptera	XM_062845937.1
Aedes aegypti	Insecta	Diptera	XM_001661937.3
Anopheles nili	Insecta	Diptera	XM_053822954.1
Culex pipiens pallens	Insecta	Diptera	XM_039593557.2
Drosophila melanogaster	Insecta	Diptera	AY079170.1
Cryptotermes secundus	Insecta	Blattodea	XM_023869322.2
Periplaneta americana	Insecta	Blattodea	XM_069816825.1
Anabrus simplex	Insecta	Orthoptera	XM_067155105.2
Schistocerca nitens	Insecta	Orthoptera	XM_049942972.1
Megachile rotundata	Insecta	Hymenoptera	XM_003704005.2
Osmia bicornis bicornis	Insecta	Hymenoptera	XM_029196857.2
Cimex lectularius	Insecta	Hemiptera	XM_014388818.2
Halyomorpha halys	Insecta	Hemiptera	XM_014415614.3
Anoplophora glabripennis	Insecta	Coleoptera	XM_018723410.2
Sitophilus oryzae	Insecta	Coleoptera	XM_030912428.1
Galleria mellonella	Insecta	Lepidoptera	XM_026905948.3
Helicoverpa zea	Insecta	Lepidoptera	XM_047173904.1
Centruroides sculpturatus	Arachnida	Scorpiones	XM_023387813.1
Dermacentor albipictus	Arachnida	Acari	XM_065453098.2
Varroa destructor	Arachnida	Acari	XM_022812260.1
Daphnia magna	Crustacea	Diplostraca	XM_032933611.2
Cherax quadricarinatus	Malacostraca	Decapoda	XM_053772963.2
Penaeus vannamei	Malacostraca	Decapoda	XM_070129803.1
Palaemon carinicauda	Malacostraca	Decapoda	XM_068373949.1
Amphibalanus amphitrite	Maxillopoda	Thoracica	XM_043357977.1
Limulus polyphemus	Merostomata	Xiphosura	XM_013939332.1

Table S4. *Ae. albopictus* CYP450 information.

CYP450 in Genebank	Gene ID	Name in Vector-base	CYP450 in Genebank	Gene ID	Name in Vector-base
CYP4AC1	AALFPA_044369	CYP4AC1	CYP6A13	AALFPA_075820	CYP6A13
CYP4C1	AALFPA_068089	CYP4C1	CYP6A14	AALFPA_062025	CYP6A14
CYP4D1	AALFPA_074802	CYP4D1	CYP6N14	AALFPA_060298	CYP6N14
CYP4D2	AALC636_022512	CYP4D2	CYP6A17	AALFPA_078634	CYP6A17
CYP4V2	AALC636_012799	CYP4V2	CYP6A20	AALFPA_062336	CYP6A20
CYP4C3	AALFPA_072108	CYP4C3	CYP9AE1	AALC636_008530	CYP9E2-like
CYP4K3	AALC636_032838	CYP4K3	CYP9J1	AALC636_034911	CYP9F2-like
CYP4D8	AALC636_029111	CYP4D8	CYP9B2	AALFPA_063118	CYP9B2
CYP4D14	AALFPA_061469	CYP4D14	CYP9E2	AALC636_000939	CYP9E2
CYP4G15	AALFPA_071364	CYP4G15	CYP9F2	AALC636_032354	CYP9F2
CYP4J15	AALFPA_045061	CYP4C1-like	CYP9J6	AALC636_026995	CYP9E2-like
CYP4J17	AALFPA_069453	CYP4C1-like	CYP9J16	AALC636_023155	CYP9F2-like
CYP4C21	AALFPA_048426	CYP4C21	CYP9J31	AALFPA_064886	CYP9E2-like
CYP4D23	AALC636_024857	CYP4D23	CYP12A4	AALFPA_066328	CYP12A4
CYP4H32	AALF012337	CYP4H32	CYP12A5	AALFPA_055400	CYP12A5
CYP4H33	AALC636_031083	CYP4D1-like	CYP18A1	AALFPA_059969	CYP18A1
CYP4D38	AALFPA_071610	CYP4D38	CYP28D1	AALFPA_079317	CYP28D1
CYP6A1	AALC636_003420	CYP6A1	CYP28A5	AALC636_018481	CYP28A5
CYP6AL1	AALFPA_055636	CYP6D5-like	CYP49A1	AALFPA_057846	CYP49A1
CYP6A2	AALFPA_061443	CYP6A2	CYP301A1	AALFPA_067032	CYP301A1
CYP6BB2	AALF003065	CYP6BB2	CYP302A1	AALFPA_078879	CYP302A1
CYP6F2	AALC636_031381	CYP6D5-like	CYP304A1	AALC636_006619	CYP304A1
CYP6G2	AALC636_036644	CYP6G2	CYP304C1	AALC636_016525	CYP304A1-like
CYP6AG3	AALC636_014096	CYP6AG3	CYP304B2	AALFPA_049004	CYP304A1-like
CYP6D3	AALFPA_060447	CYP6D3	CYP305A1	AALFPA_055450	CYP305A1
CYP6F3	AALFPA_066064	CYP6D5-like	CYP305A5	AALC636_008425	CYP305A1-like
CYP6N3V3	AALFPA_073654	CYP6N3V3	CYP306A1	AALFPA_075327	CYP306A1
CYP6S3	AALC636_021295	CYP6A20-like	CYP307A1	AALFPA_045049	CYP307A1
CYP6D4	AALFPA_052037	CYP6D4	CYP308A1	AALFPA_053582	CYP308A1
CYP6D5	AALC636_014074	CYP6D5	CYP313A4	AALFPA_073978	CYP313A4
CYP6AG6	AALC636_012310	CYP6A8-like	CYP314A1	AALF004962	CYP314A1
CYP6M6	AALC636_023635	CYP6M6	CYP315A1	AALFPA_079547	CYP315A1
CYP6N6	AALC636_038277	CYP6A14-like	CYP325L1	AALFPA_069453	CYP4C1-like
CYP6Z6	AALC636_018232	CYP6D3-like	CYP325N1	AALC636_018086	CYP4C1-like
CYP6AG7	AALF015756	CYP6AG7	CYP325T1	AALF015449	CYP4C21-like
CYP6A8	AALC636_036378	CYP6A8	CYP325X1	AALFPA_080571	CYP4C21-like
CYP6Z8	AALC636_025939	CYP6D3-like	CYP325G2	AALFPA_042344	CYP4C1-like
CYP6N9	AALFPA_075999	CYP6A14-like	CYP325Q2	AALC636_008893	CYP4C21-like
CYP6M10	AALC636_021415	CYP6A14-like	CYP325K3	AALC636_022629	CYP4C1-like
CYP6N11	AALC636_006654	CYP6A13-like	CYP325S3	AALFPA_057175	CYP4C21-like
CYP6N12	AALC636_026235	CYP6A14-like			

Table S5. *Ae. albopictus* endocytic genes and related sequences.

Ae. albopictus		Orthologues			
**Gene**	**Gene ID**	***Ae. aegypti***	**% Identity**	***D. melanogaster***	**% Identity**
AP50	AALC636_033072	AAEL003106	88.85	CG7057	83.18
Chc	AALC636_022815	AAEL022819	89.46	CG9012	76.76
lqf	AALC636_033617	AAEL009088	91.30	CG8532	73.35
lqfR	AALFPA_060726	AAEL013724	84.20	CG42250	80.58
Dynamin	AALFPA_067314	AAEL007288	89.69	CG18102	73.61
Vha16	AALC636_000071	AAEL000291	85.29	CG3161	82.35
VhaSFD	AALFPA_048766	AAEL006516	85.27	CG17332	77.08
Rab7	AALC636_020391	AAEL003503	84.14	CG5915	76.44
Arf72	AALFPA_076650	AAEL014177	81.90	CG6025	80.80
Flot-1	AALFPA_080924	AAEL025017	88.88	CG8200	78.49
Flot-2	AALC636_000218	AAEL004041	88.16	CG32593	78.25

Table S6. The number of genera and OTUs in different groups of phytoplankton at the phylum level.

Phytoplankton	JNL		JNL-HOC5		HOC5-314A		HOC5-315A	
	**Genus**	**OTUs**	**Genus**	**OTUs**	**Genus**	**OTUs**	**Genus**	**OTUs**
Chlorophyta	28	120	27	126	30	124	27	112
Chrysophyta	15	72	17	63	15	61	16	63
Cryptophyta	7	56	9	57	9	63	8	56
Bacillariophyta	13	48	11	41	15	56	12	46
Pyrrophyta	6	33	6	33	6	31	5	28
Streptophyta	8	22	9	25	10	32	6	20
Protalveolata	2	28	2	28	2	26	2	22
Ochrophyta	4	11	5	8	5	11	4	7
Phragmoplastophyta	1	3	1	1	1	3	1	1
Glaucophyta	1	1	1	1	1	1	1	1
Haptophyta	0	1	0	1	0	1	0	0
Total	85	395	88	384	94	409	82	356

Table S7. The number of genera and OTUs in different groups of zooplankton at the phylum level.

Zooplankton	JNL		JNL-HOC5		HOC5-314A		HOC5-315A	
	**Genus**	**OTUs**	**Genus**	**OTUs**	**Genus**	**OTUs**	**Genus**	**OTUs**
Ciliophora	41	154	42	159	46	169	44	134
Cercozoa	4	28	3	29	4	33	4	28
Arthropoda	5	15	4	16	5	30	5	15
Rotifera	2	12	3	13	3	10	2	7
Heliozoa	3	7	3	7	3	7	3	7
Chordata	1	7	1	8	1	12	1	5
Gastrotricha	0	2	0	2	0	2	0	1
Mollusca	0	2	2	7	0	8	1	4
Nematoda	0	2	0	0	1	2	0	0
Platyhelminthes	1	1	1	1	1	1	1	1
Bryozoa	1	1	0	0	0	0	1	1
Annelida	0	1	0	1	0	1	0	0
Apicomplexa	0	0	0	0	1	2	0	0
Total	58	232	59	243	65	277	62	203

MAST is an acronym that stands for 'MArine STramenopiles'. It was originally coined in marine plankton research to refer to a diverse group belonging to the phylum Stramenopiles (unequal flagellates).

Table S8. Comparison of sequence homology between *Ae. albopictus cyp315a1* dsRNA region and zooplankton target genes.

Ae. albopictus **CYP315A1 specific sequence 5′-3′**	**Zooplankton species**	**Target gene name**	**Gene ID**	**homologous rate**
CGAAAAGCATAAATGCGAAC-GAGG	Strombidium sp.	small subunit ribosomal RNA	MZ687717.1	80.84%
AAAGATCCTGAAATGATGA-GAAG<br>CGGTAAGGCTCGACCTTTCAAA	Stokesia laevis	1.internal transcribed spacers<br>2.trnL gene	AH007083.2<br>AH007249.2	79.57%<br>68.19%
CAACGAAAA-GCATAAATGCGAACGAGGCCT	Ichthyobodo sp.	18S ribosomal RNA	AY255800.1	80%
ATA-TACTGAAGTCTCACATTAAATT	Bryometopus atypicus	Brat1 small subunit ribosomal RNA	HM246401.1	78.00%
TATTCAAAATTAAAA-TAGCTAACGT<br>AGGCCTATTTTTTATGGACGAT	Cryptocaryon irritans	x99 ribosomal protein L11<br>elongation factor-1 alpha isoform-3	JX103970.1<br>AB275857.1	83%<br>82.73%
TCTTAAAA-GATCCTGAAATGATGAGAAG<br>AAAAGATCCTGAAATGATGA-GAAG	Halteria sp.	ZH-9 large subunit ribosomal RNA<br>ZJ-11 large subunit ribosomal RNA	MK501392.1<br>MK501432.1	87.86%<br>83.34%
CAGTATTCAAAATTAAAATAGC	Dileptus nasutum	KTC14 small subunit ribosomal RNA	KY855551.1	78.19%
AATTAAAATAGCTAAC-GTAAATGC	Paracercomonas Sp.	XT179 internal transcribed spacer	FJ790736.1	76.93%
ATAAATGCGAACGAGGCC-TATTTTTTATG	Ophryoscolex caudatus	[Fe]-hydrogenase	AM396957.1	82.42%

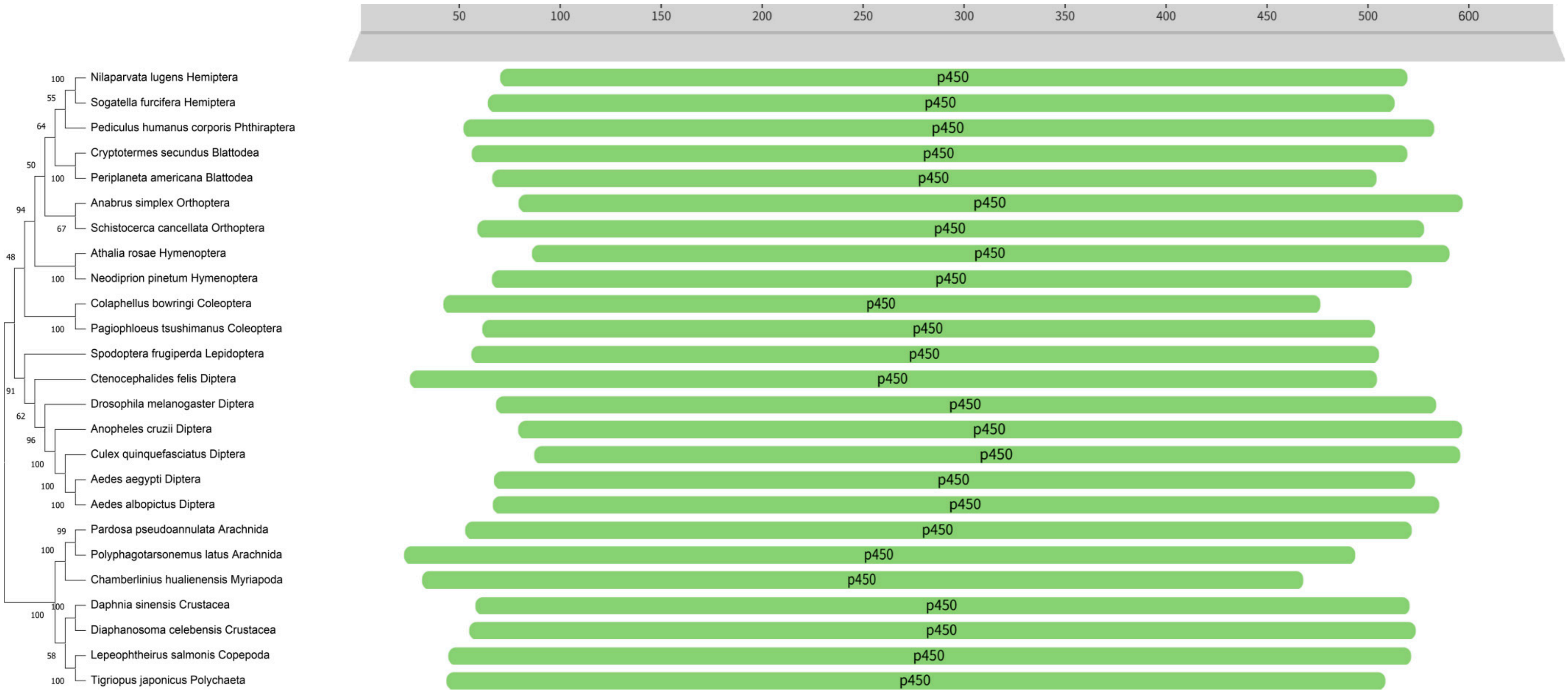

Figure S1. Phylogenetic relationship of CYP314A1 and analysis of their conserved domains. Phylogenetic relationship of CYP314A1 from Arthropoda. A Neighbor-Joining (NJ) tree was constructed using MEGA11 based on the alignment of CYP314A1 protein sequences. Bootstrap analysis was performed with 1000 replicates. The numbers are bootstrap values based on 1000 iterations. (B) The domains of each CYP314A1 protein were predicted using the online InterProScan tool (http://www.ebi.ac.uk/InterProScan). The p450 region represents the functional regions of CYP314A1.

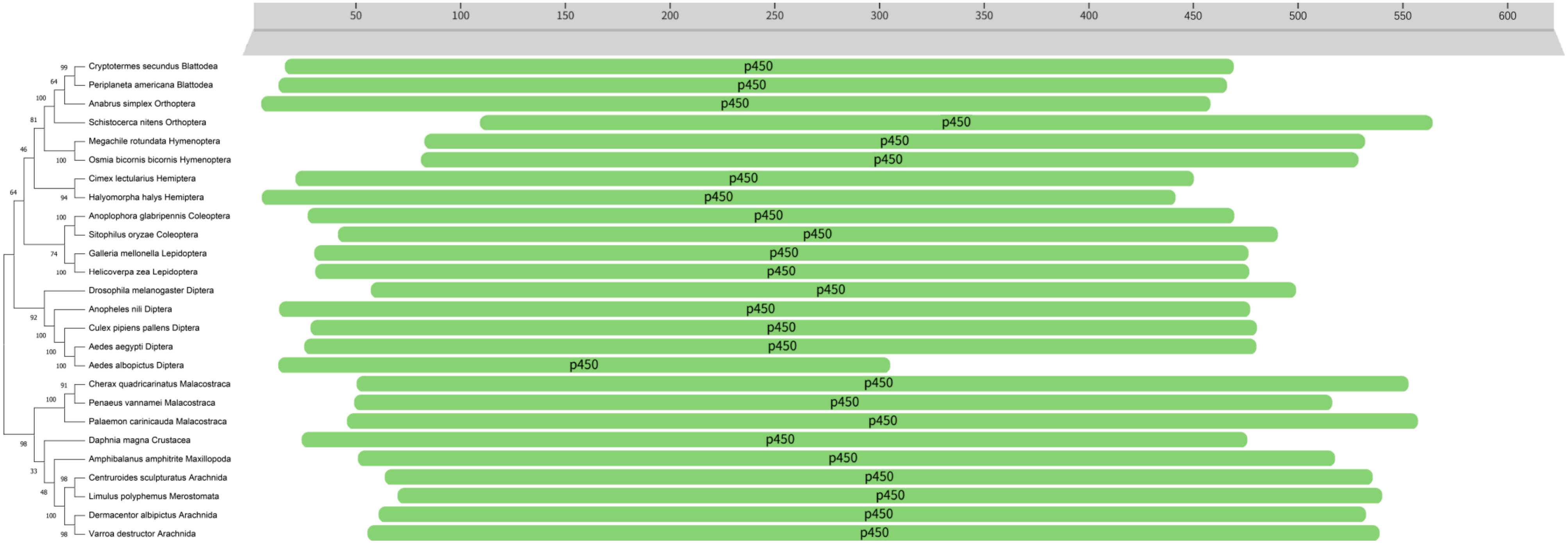

Figure S2. Phylogenetic relationship of CYP315A1 and analysis of their conserved domains. (A) Phylogenetic relationship of CYP315A1 from Arthropoda. A Neighbor-Joining (NJ) tree was constructed using MEGA11 based on the alignment of CYP315A1 protein sequences. Bootstrap analysis was performed with 1000 replicates. The numbers are bootstrap values based on 1000 iterations. (B) The domains of each CYP315A1 protein were predicted using the online InterProScan tool (http://www.ebi.ac.uk/InterProScan). The p450 region represents the functional regions of CYP315A1.

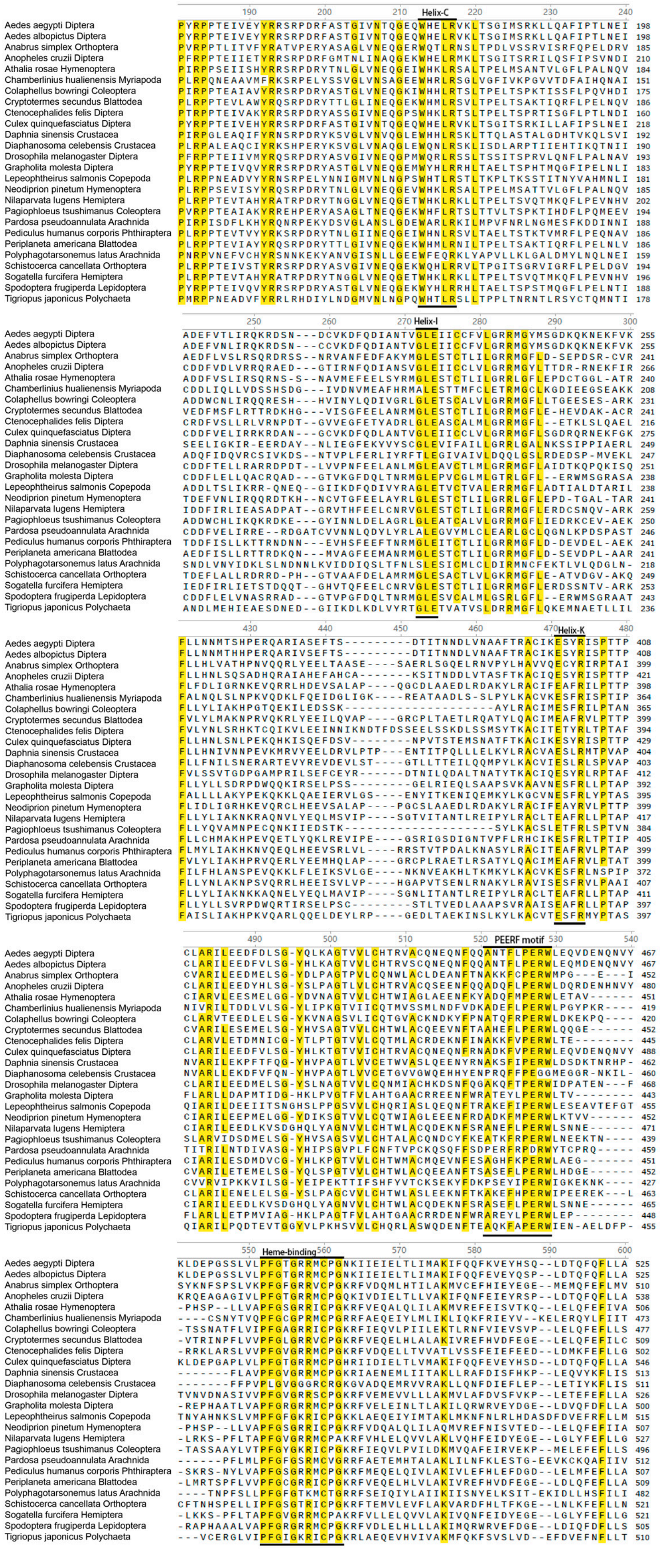

Figure S3. The alignment of the amino acid sequences of CYP314A1 in *Ae. albopictus* and its orthologues in the Arthropoda. The black lines represent the Helix-C (WxxR), Helix-K (ExxR), Helix-I (GxR/DTT/S), PERF(PxxFxPE/DRF) and Heme-binding (PFxxGxRxG/A) domains, respectively, where 'x' represents any amino acid.

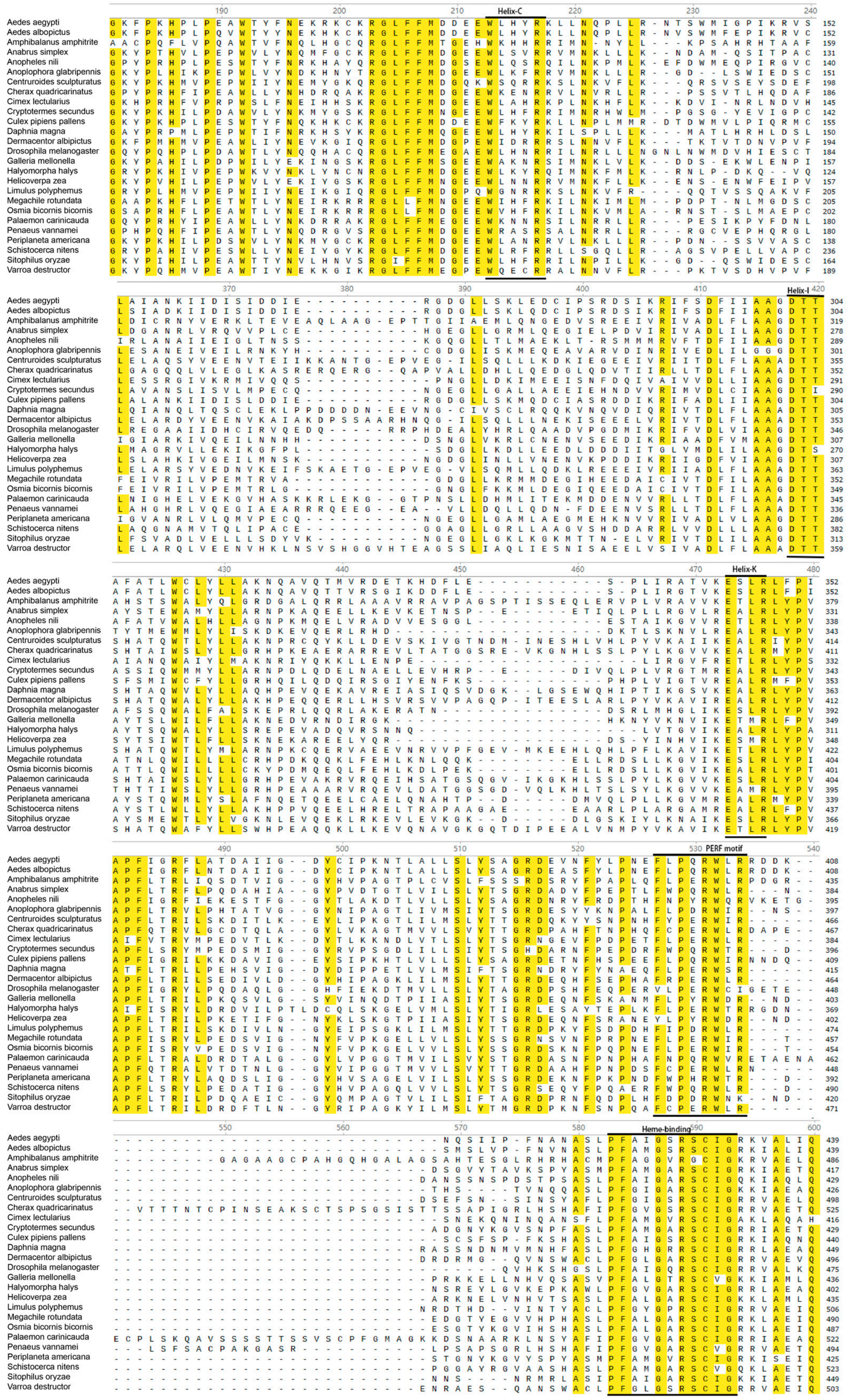

Figure S4. The alignment of the amino acid sequences of CYP315A1 in *Ae. albopictus* and its orthologues in the Arthropoda. The black lines represent the Helix-C (WxxR), Helix-K (ExxR), Helix-I (GxR/DTT/S), PERF(PxxFxPE/DRF) and heme-binding (PFxxGxRxG/A) domains, respectively, where 'x' represents any amino acid.

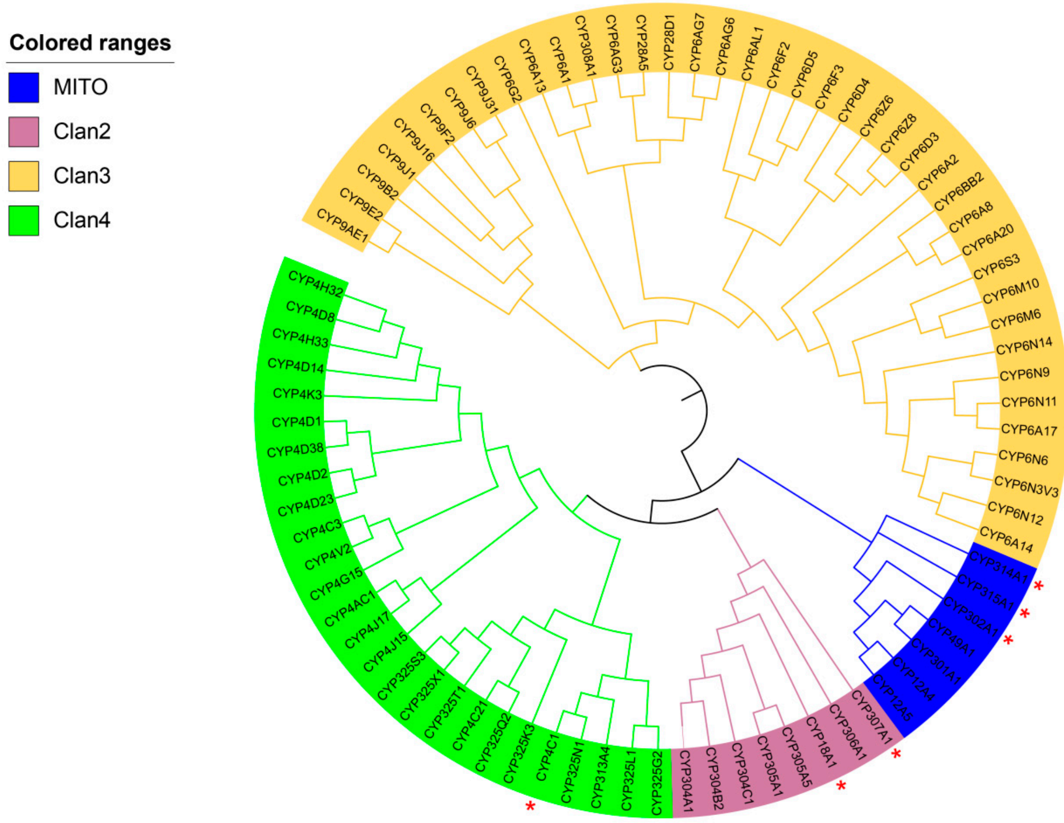

Figure S5. Phylogeny of *Ae. albopictus* cytochrome P450. CYP450 protein sequences were used to create a circular diagram showing clusters of related CYP450s. As can be seen, individual clans are represented by different colors. Key proteins involved in the ecdysteroid synthesis pathway are marked with red asterisks.

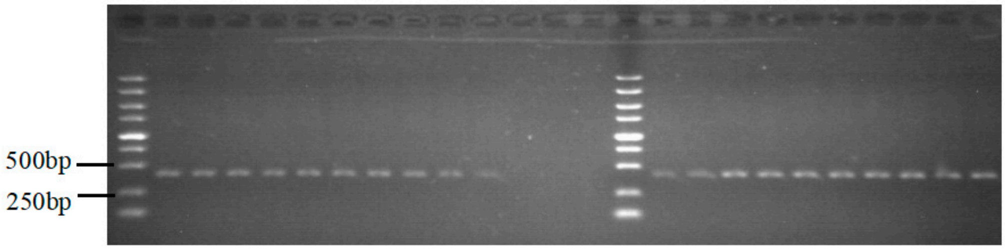

A B

Figure S6. PCR electrophoresis results of recombinant plasmid pMaa7IR/cyp314a1IR(A) and pMaa7IR/cyp315a1IR (B) transgenic algal lines. (A) M: DL1000 DNA marker; 1-5: partial PCR product of pMaa7IR/cyp314a1IR transformed *Chlamydomonas* lines; 6-10: partial PCR product of pMaa7IR/cyp314a1IR transformed *Chlorella* lines. (B) M: DL1000 DNA marker; 1-5: Partial PCR results of pMaa7 IR/cyp315a1IR transformed *Chlamydomonas;* 6-10: Partial PCR results of pMaa7IR/cyp315a1IR transformed *Chlorella* lines. The primers were designed with the promoter region of the pMaa7IR/XIR vector, and the resulting amplification product was 430 bp.

***cyp314a1* shRNA**

CCGGACGGAAATCGTGGAGTATTATCTCGAGATAATACTCCACGATTTCCGTTTTTG

***cyp315a1* shRNA**

CCGGATACTGAAGTCTCACATTAAACTCGAGTTTAATGTGAGACTTCAGTATTTTTG

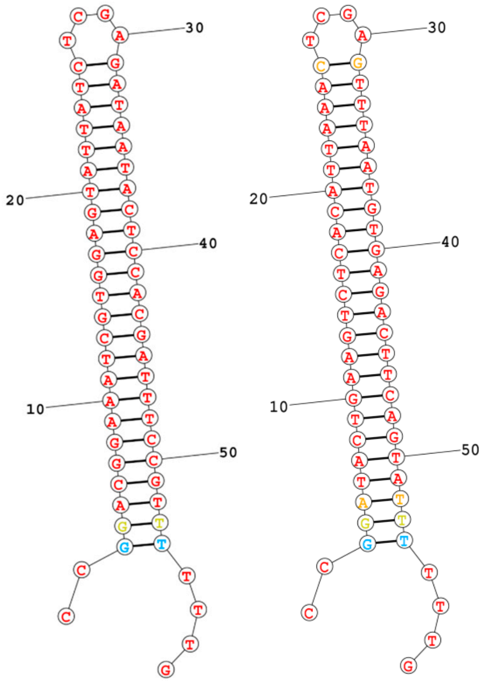

cyp314a1 cyp315a1

Figure S7. Sequence of *cyp314a1* and *cyp315a1* shRNA and predicted shRNA secondary structure. The shRNA targeting cyp314a1/CYP315a1 was designed using the online platform https://portals.broadinstitute.org/gpp/public/ and its secondary structure was predicted using the online tool http://www.unafold.org/. The synthesized shRNAs (BGI Genomics Co., Ltd., Shenzhen, China) were cloned into the pCAMBIA1302 expression vector. The final constructs, pCAMcyp314a1-shRNA and pCAMcyp315a1-shRNA, were verified by sequencing.
